# A Comprehensive Investigation of Methanol Electrooxidation on Copper Anodes: Spectroelectrochemical Insights and Energy Conversion in Microfluidic Fuel Cells

**DOI:** 10.1021/acsami.4c08472

**Published:** 2024-06-25

**Authors:** Breno D. Queiroz, Pedro-Lucas S. Vital, Kaê O. Budke, Natalia Rey-Raap, Ana Arenillas, Guilherme M. O. Barra, Dênis S. Ferreira, Giuseppe A. Camara, Heberton Wender, Cauê A. Martins

**Affiliations:** † Institute of Physics, Universidade Federal de Mato Grosso do Sul, CP 549, Campo Grande, MatoGrosso do Sul 79070-900, Brazil; ‡ Group MATENERCAT, Instituto de Ciencia y Tecnología del Carbono, INCAR-CSIC, Francisco Pintado Fe 26, Oviedo 33011, Spain; § Departamento de Engenharia Mecânica, Universidade Federal de Santa Catarina, Florianópolis 88040-900, Brazil; ∥ Institute of Chemistry, Universidade Federal de Mato Grosso do Sul, CP 549, Campo Grande, MatoGrosso do Sul 79070-900, Brazil

**Keywords:** copper anode, metal-free cathode, FTIR *in situ*, fuel cell, energy conversion

## Abstract

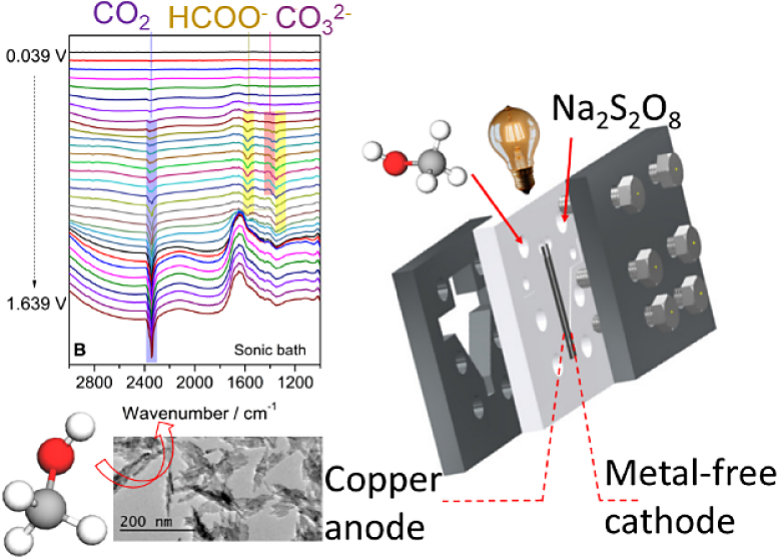

Here, we comprehensively investigated methanol electrooxidation on Cu-based catalysts, allowing us to build the first microfluidic fuel cell (μFC) equipped with a Cu anode and a metal-free cathode that converts energy from methanol. We applied a simple, fast, small-scale, and surfactant-free strategy for synthesizing Cu-based nanoparticles at room temperature in steady state (ST), under mechanical stirring (MS), or under ultrasonication (US). The morphology evaluation of the Cu-based samples reveals that they have the same nanoparticle (NP) needle-like form. The elemental mapping composition spectra revealed that pure Cu or Cu oxides were obtained for all synthesized materials. In addition to having more Cu_2_O on the surface, sample US had more Cu(OH)_2_ than the others, according to X-ray diffractograms and X-ray photoelectron spectroscopy. The sample US is less carbon-contaminated because of the local heating of the sonic bath, which also enhances the cleanliness of the Cu surface. The activity of the Cu NPs was investigated for methanol electrooxidation in an alkaline medium through electrochemical and spectroelectrochemical measurements. The potentiodynamic and potentiostatic experiments showed higher current densities for the NPs synthesized in the US. *In situ* FTIR experiments revealed that the three synthesized NP materials eletcrooxidize methanol completely to carbonate through formate. Most importantly, all pathways were led without detectable CO, a poisoning molecule not found at high overpotentials. The reaction path using the US electrode experienced an additional round of formate formation and conversion into carbonate (or CO_2_ in the thin layer) after 1.0 V (vs. Ag/Ag/Cl), suggesting improved catalysis. The high activity of NPs synthesized in the US is attributed to effective dissociative adsorption of the fuel due to the site’s availability and the presence of hydroxyl groups that may fasten the oxidation of adsorbates from the surface. After understanding the surface reaction, we built a mixed-media μFC fed by methanol in alkaline medium and sodium persulfate in acidic medium. The μFC was equipped with Cu NPs synthesized in ultrasonic-bath-modified carbon paper as the anode and metal-free carbon paper as the cathode. Since the onset potential for methanol electrooxidation was 0.45 V and the reduction reaction revealed 0.90 V, the theoretical OCV is 0.45 V, which provides a spontaneous coupled redox reaction to produce power. The μFC displayed 0.56 mA cm^–2^ of maximum current density and 26 μW cm^–2^ of peak power density at 100 μL min^–1^. This membraneless system optimizes each half-cell individually, making it possible to build fuel cells with noble metal-free anodes and metal-free cathodes.

## 1 Introduction

The search for sustainable energy converters for portable electronics led to the development of miniaturized devices called microfluidic fuel cells (μFCs).^1,2^ The μFCs produce an electric current through a spontaneous redox coupled reaction. Namely, the electrooxidation of fuel takes place at the anode while an oxidant is electroreduced at the cathode side—these reactions continuously conduct electrons through an external circuit, which is converted into electric work. The main advantage of the μFCs is the absence of an anionic exchange membrane, so the price is reduced, excluding the membrane’s intrinsic ohmic drop.^1^ The membrane is replaced by the colaminar flow, built by conducting the two flows—anolyte and catholyte—in low Reynolds numbers toward the outlet.^2^ The thin film built by the colaminar flow is responsible for the ionic exchange and internal electric connection between the two sides.

One of the most addressed challenges to decreasing the overall price and improving the performance of these devices is partially/totally replacing Pt catalysts used at the anode and cathode.^3−7^ Although a promising strategy for microbial fuel cells^8,9^ and paper-based μFCs,^10^ the use of non-noble metals at the anode side (or of metal-free anodes) delays the onset potential of the reaction, which narrows the difference between the reduction potential (*E*_cathode_) and the oxidation potential (*E*_anode_). This effect makes the overall reaction less spontaneous since Δ*G* = −*nFE*, where Δ*G* is the free Gibbs energy of the overall reaction, *F* is the Faraday constant, and *E* = *E*_cathode_ – *E*_anode_. However, μFCs permit an unexplored alternative to increase *E* by independently decreasing *E*_anode_ and increasing *E*_cathode_—the use of mixed media.^11^

The absence of a membrane allows using anolytes and catholytes with independent pHs.^12−14^ Thus, it is possible to shift the Nernstian potential toward lower values at the anode by using an alkaline medium and toward higher values at the cathode by using acidic solutions. This configuration may be the key to non-noble-metal catalysts, since the open-circuit voltage (OCV) can be broadened, making the overall reaction more spontaneous (Δ*G* more negative).

Copper is a cheap and widely available non-noble metal that could be exploited as an anode for methanol electro-oxidation in an alkaline medium. Copper has been investigated as a sole catalyst and as cocatalyst for methanol electrooxidation.^15−22^ However, at least under our concern, only one work investigates the reactions’ pathways or mechanisms.^23^ Therefore, understanding this half-cell reaction is still necessary to improve its use in a fuel cell.

This reaction can be coupled to hypochlorous acid reduction,^12,14,24^ or persulfate^25^ reduction reactions. These reactants have been proven to be outstanding liquid oxidants.^26^ Most importantly, the use of liquid oxidants decreases *starving* at the cathode and opens up the possibility of using metal-free cathodes, where carbon paper is directly used as cathodes.^24−26^

Here, we synthesized Cu nanoparticles (NPs) in a new reduced-scale chemical protocol, controlling the mass diffusion by stirring and an ultrasonic bath compared to the stationary system. These Cu NPs were characterized and investigated toward methanol electrooxidation by electrochemical and spectroelectrochemical measurements to reveal new insights into the understanding of the reaction. The Cu NPs were dispersed on carbon paper (CP) and used as the anode, while CP was directly used as the cathode in a methanol/sodium persulfate mixed-media 3D-printed microfluidic fuel cell.

## 2 Experimental Section

### 2.1 Synthesis and Characterization of the Cu Nanoparticles

The synthesis of the Cu NPs was adapted from the synthesis of Quinson et al.^27^ Briefly, the cationic Cu from the precursor was aged and reduced in stationary conditions, under stirring, and in an ultrasonic bath in methanol solution. The precursor H_2_CuCl_4_ was prepared by adding 0.0852 g of Cl_2_Cu·2H_2_O in 10 mL of 0.2 mol L^–1^ methanol in HCl. Next, this solution was transferred to a 25 mL volumetric flask, filled with methanol, and aged for 48 h. Another solution was prepared by adding 1.25 mL of methanol and 21.4 mg of LiOH·H_2_O (98%) in 10 mL of deionized water. Next, this solution was aged for 24 h.

The reduced scale synthesis was performed in 2.0 mL Eppendorf tubes by simply mixing 1.75 mL of methanolic LiOH solution with 0.25 mL of the H_2_CuCl_4_ precursor solution. Pictures in Figure S1 illustrate the advances in the reaction degree. The (i) synthesis in stationary (ST) condition was finished after 24 h in steady-state when it was placed in the fridge at −2 °C. Another synthesis was performed (ii) for 24 h in mechanical stirring (MS) at 1250 rpm until put in the fridge. The third protocol followed (iii) sonicating (US) the sample during the first 3 h, leaving it steady for 24 h, and putting it in the fridge.

After 24 h in the refrigerator, the Cu NPs were sonicated for 6 min and centrifuged for 1 h in an Eppendorf centrifuge at 6000 rpm. The supernatant was discarded, and the Eppendorf tube was completed with deionized water. This protocol was followed three times. Finally, the samples were diluted in 1 mL of water, stored at room temperature, and protected from light.

The morphology of the samples was examined using a scanning electron microscope (SEM, JEOL model JSM-6380LV) equipped with an energy-dispersive X-ray spectrometer (EDS) Thermo Scientific (Noran System Six). For these analyses, we dropped the preultrasonicated NP solutions on silicon plates. Also, we prepared 19 mm × 1 mm carbon paper (CP) samples that were immersed in the Cu NP aqueous suspension of each synthesis and left in a sonic bath for 5 min before drying and analysis by SEM and EDS. A transmission electron microscope (TEM, JEOL 2100Plus) was employed to examine dried Cu NPs’ morphology.

Powder X-ray diffraction (XRD) was performed by using Cu Kα radiation on a D8 ADVANCED diffractometer to study the phase structure and crystallinity of the nanoparticles. The data were collected over a 2θ range from 20.00 to 80.00° with a step size of 0.05°. The porous properties were characterized by Ar adsorption–desorption isotherms at 87 K in an adsorption analyzer (Micromeritics ASAP 2020) after degassing the samples at 120 °C and 0.1 mbar for 8 h using a degasser (Micromeritics VacPrep 061). The surface chemical composition was analyzed by X-ray photoelectron spectroscopy (XPS) using a Kratos AXIS Ultra HAS, with a monochromatic Al Kα X-ray source (1486.7 eV) with a pass energy of 30 eV for high-resolution regions of interest and 100 eV for the survey. Samples were fixed at the sample holder using carbon tape.

The masses of metallic catalysts on CP for the three types of catalysts were investigated by thermogravimetry. We used TGA Q50 V20.13 Build 39 equipment communicating to Universal V4.5A TA Instruments software to heat the samples from room temperature to 900 at 20 °C min^-1^ under synthetic air. A simple difference between the initial and final mass was used to infer the mass of Cu on CP.

### 2.2 Electrochemical and Spectroelectrochemical Measurements

The electrochemical measurements were performed in a conventional three-electrode glass electrochemical cell. A high-area Pt plate was used as a counter electrode and Ag/AgCl as a reference. A mirror-polished and cleaned glassy carbon modified with Cu NPs was used as the working electrode. For that, the Eppendorf tube was sonicated for a few seconds, and 50 μL were placed onto a 0.2 cm^2^ glassy carbon electrode and dried under an N_2_ atmosphere. All electrochemical measurements were performed in a PalmSens 3. The profiles of the NPs were collected in 0.1 mol L^–1^ O_2_-free KOH between −0.9 and 0.7 V vs. Ag/AgCl at 0.05 V s^–1^. The electrocatalytic parameters were collected in 0.5 mol L^–1^ methanol + 0.1 mol L^–1^ O_2_-free KOH between −0.4 and 1.6 V vs. Ag/AgCl at 0.05 V s^–1^. The chronoamperometric experiments were performed by applying 0.6 V for 1800 s.

Methanol electrooxidation reaction was investigated by *in situ* Fourier-transform infrared (FTIR) using a PerkinElmer (model Frontier) spectrometer with an MCT (mercury–cadmium–telluride) detector cooled with liquid nitrogen. The spectroelectrochemical cell comprises a Pt tape counter electrode, Ag/AgCl reference, and a modified glassy carbon working electrode, sealed with a CaF_2_ window connected to a Potentiostat PalmSens (Model PSTrace 4). The spectra were collected from 4000 to 1000 cm^–1^ at 8 cm^–1^ resolution during voltammograms between 0.039 and 1.639 V vs. Ag/AgCl at 1 V s^–1^ in 0.5 mol L^–1^ methanol + 0.1 mol L^–1^ O_2_-free KOH. The spectra were calculated as the change in reflectance compared to the reference spectrum obtained at 0.039 V.

The cathodic reaction was also investigated in half-cell measurements. A roughly 2 mm × 12 mm carbon paper (060 Toray) foil was investigated in an O_2_-free 1.0 mol L^–1^ sodium persulfate (Na_2_S_2_O_8_) in +1.0 mol L^–1^ H_2_SO_4_ by cyclic voltammetry between 0.2 and 1.8 V vs. Ag/AgCl at 0.05 V s^–1^.

### 2.3 Additive Manufacturing of the Microfluidic Fuel Cell

**Figure 1:**
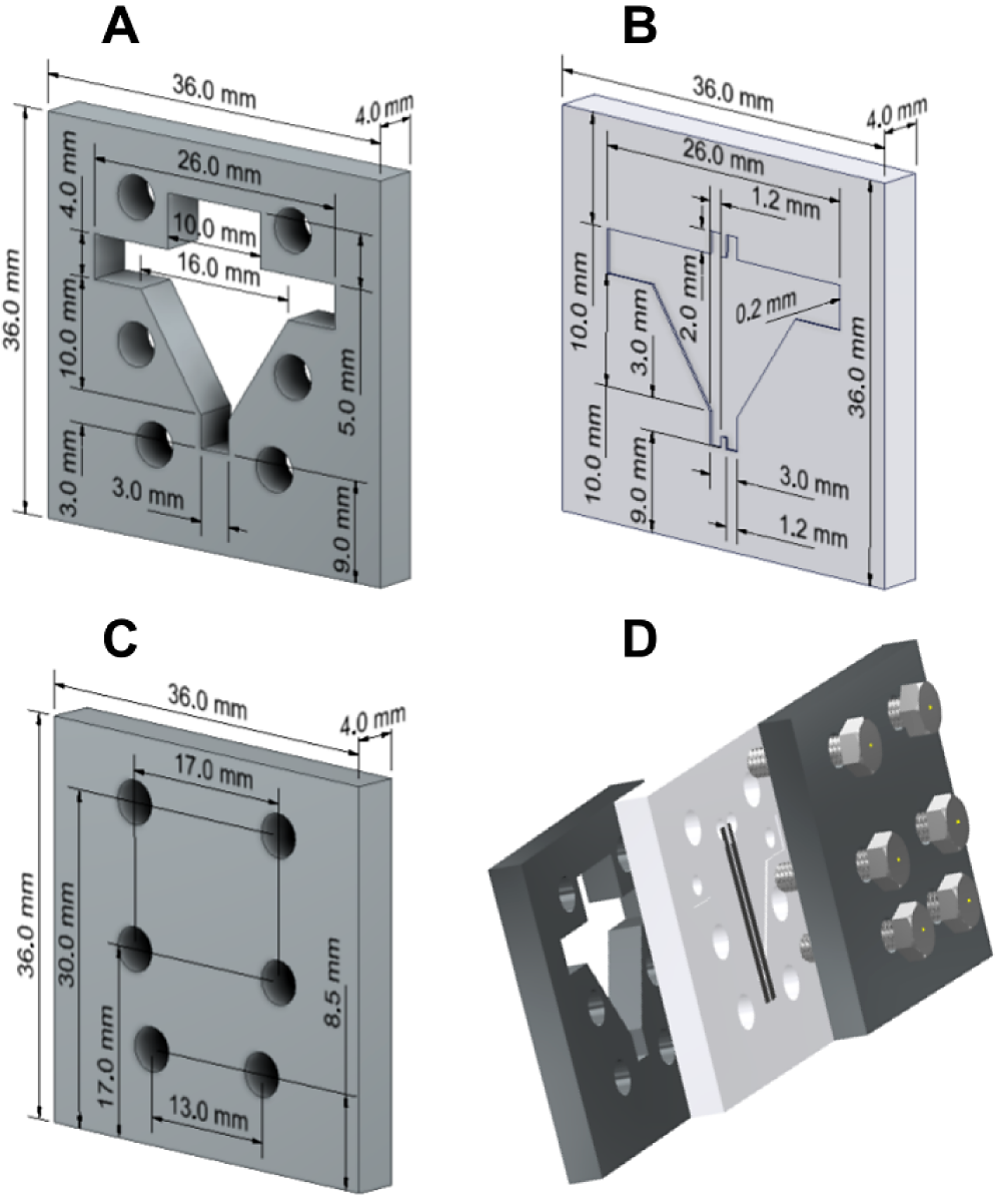
Illustrative scheme of the membraneless microfluidic fuel cell, featuring (A) the printed top part, (B) the PDMS part containing the microchannel, (C) the printed bottom part, and (D) a disassembled cell.

Based on previous work, we prototyped a reusable microfluidic fuel cell with 3D-printed pieces and a polydimethylsiloxane (PDMS) channel.^28^ The 3.6 cm × 3.6 cm cell is composed of these two printed pieces, one with the two inlets on top (Figure 1A) and another smooth plate at the bottom (Figure 1C), sandwiching a 4-mm thick PDMS piece containing the colaminar channel (Figure 1B). The porous electrodes (modified and unmodified carbon papers) are placed on the grooves of the PDMS (Figure 1D), forming a cross-sectional area of 0.0266 cm^2^, which is used for current and power normalization in fuel cell tests. The porous electrodes are connected to a thin copper plate by silver epoxy and then to the potentiostat. The cell operates with a stable colaminar flow. The microchannel is 200 μm high, and the CP is 190 μm. At this configuration, part of the liquid goes throughout the cell intact but part goes through the porous electrodes, increasing the collision factor.

The top (Figure 1A) and bottom (Figure 1C) parts were printed using a poly(lactic acid) filament (PLA) by fused deposition modeling (FDM) using a 3D-Printer Sethi3D. The model was sliced using Simplify 3D software. A positive template was printed to cure the negative PDMS piece. After assembling this μFC with the Cu/CP anode and CP cathode using six screws and nuts, the cell was tested in a methanol/persulfate mixed-media system.

### 2.4 Microfluidic Fuel Cell Tests

The fuel cell tests were performed with the assembly described in Figure 1D, with a Cu/CP anode fed by O_2_-free 0.5 mol L^–1^ methanol +0.1 mol L^–1^ KOH as the anolyte and metal-free CP cathode fed by 1.0 mol L^–1^ Na_2_S_2_O_8_ + 1.0 mol L^–1^ H_2_SO_4_ catholyte. Here, we used the Cu NPs synthesized in an ultrasonic bath due to the high activity and efficient conversion shown in half-cell and spectroelectrochemical measurements, as discussed later. The anode was prepared by immersing a 19 mm × 1 mm CP solution in a 25 mg mL^–1^ Cu aqueous suspension in a sonic bath for 5 min and dried in oven at 50 °C for 6 h. This protocol developed by Caneppele et al. improves particle dispersion.^29^ It is worth noting that we used Cu directly dispersed on CP instead of previously building an ink with Cu on a carbon support such as carbon black. This strategy aims to eliminate the adherence to the carbon support/CP, allowing us to better control the experiment.

The performance of these mixed-media systems was analyzed in terms of polarization and power density curves with liquids driven by a dual syringe pump (KDS Legato, model 101) at 50 and 100 μL min^–1^. All polarization measurements were performed with a potentiostat/galvanostat (PalmSens 3), recording the current response from the OCV to 0.01 V at a scan rate of 0.01 V s^–1^.

## 3 Results

### 3.1 Physical-Chemical Characterization

**Figure 2:**
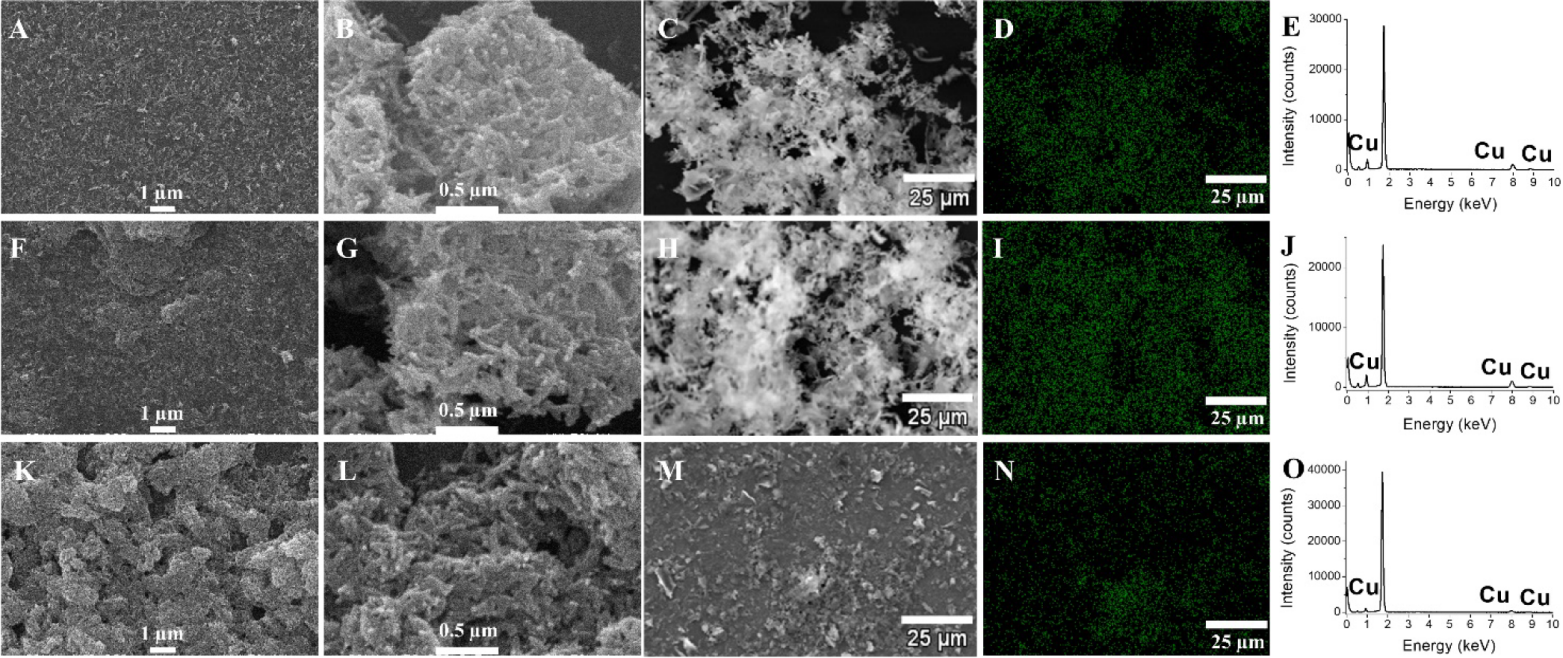
SEM images at different magnifications, EDS elemental mapping images, and EDS spectra of dried Cu NPs synthesized (A–E) in stationary conditions, (F–J) in a sonicating bath, and (K–O) with mechanical stirring.

The morphology and chemical composition of the synthesized NPs were investigated by SEM-EDS and TEM analysis. Figure 2 shows the SEM images of the Cu NPs. In general, the Cu particles synthesized in stationary (Figure 2A–C), in a sonication bath (Figure 2G–H), and under mechanical stirring (Figure 2K–M) seem similar, building needle-like particles. The elemental mapping composition (Figure 2D, N) and their respective EDS spectra (Figure 2E, J, O) show that the materials are pure copper without contamination signals.

**Figure 3:**
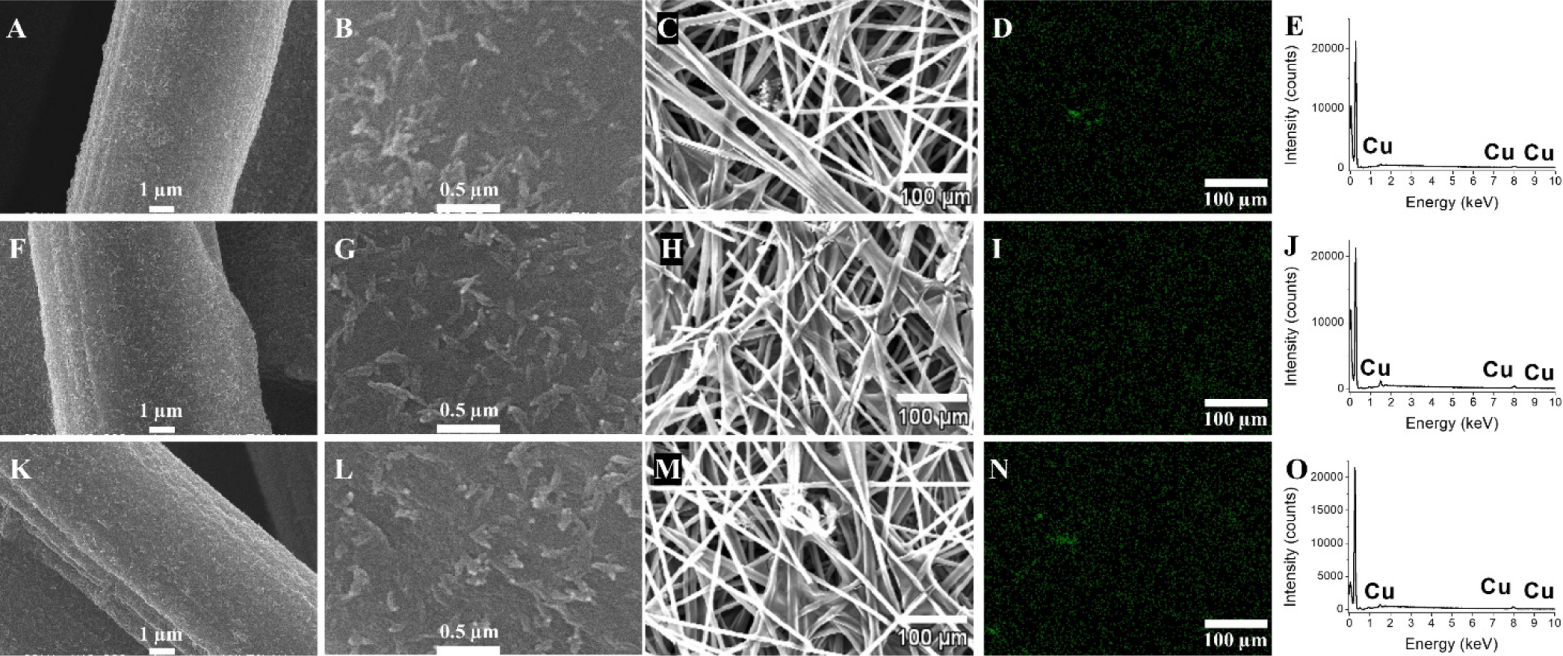
SEM images at different magnifications, EDS elemental mapping images, and EDS spectra of carbon paper-modified Cu NPs synthesized (A–E) in a stationary condition, (F–J) sonicating bath, and (K–O) mechanical stirring.

We also investigated the morphology of the Cu catalysts after dispersion on CP, as shown in Figure 3. Regardless of the synthesis method, the ultrasound-induced strategy for electrode preparation^29^ led to well-distributed NPs, as seen in the SEM images of Figure 3A, F, K. The SEM images in Figure 3B,G,L show no aggregation over the carbon fibers. The carbon fibers of the CP are permeable without clogged channels (Figure 3C, H, M), which is imperative to build a stable colaminar flow and maximize reactant utilization throughout the electrodes. Moreover, well-distributed Cu NPs increase the electrochemically active surface area. The elemental mapping composition (Figure 3D, I, N) and their EDS spectra (Figure 3E, J, O) evidence the presence of Cu, but with lower intensity peaks at the spectra compared to those for unsupported NPs (Figure 2), which is expected for an electrode with a suitable catalysts’ distribution on the carbon fibers.

**Figure 4:**
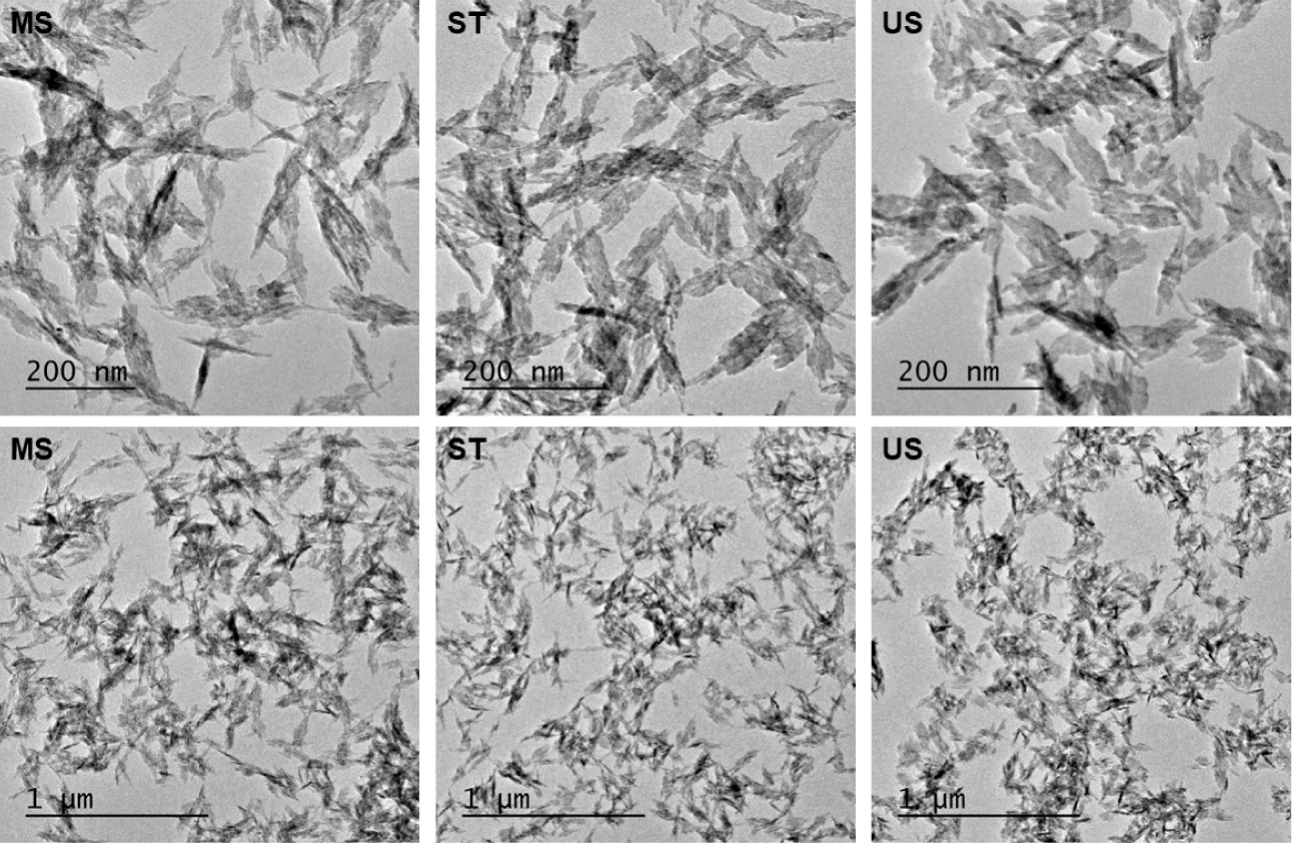
TEM images at different magnifications of samples synthesized mechanically stirred (MS), stationary (ST), and under an ultrasonic bath (US).

**Figure 5:**
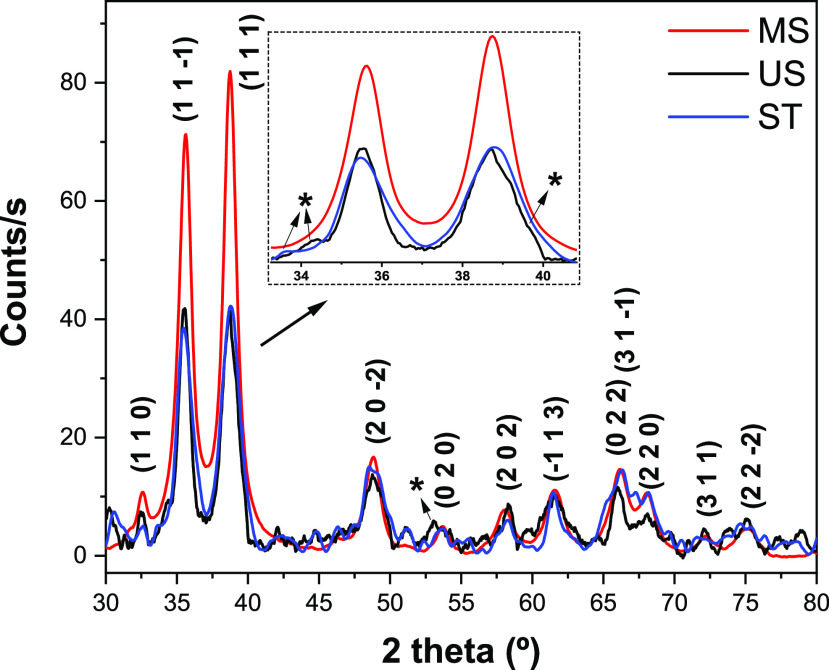
XRD patterns of samples synthesized under mechanical stirring (MS), stationary (ST), and an ultrasonic bath (US). The inset represents a zoomed area in the 34–40° region. Asterisks (*) represent Cu(OH)_2_ peaks.

Figure 4 shows the TEM images at different magnifications of Cu NPs synthesized by the three methods. These images make clear the needle-like shape of the NPs, organized in dendrites. The dendrites are ∼10 nm × 150 nm and seem only ∼2–5 nm thick. Regardless of the synthesis method, they are similar, but the NPs’ organization may induce different crystallography. To shed light on that, we performed X-ray diffractometry, shown in Figure 5.

Figure 5 shows diffractograms of samples ST, MS, and US. Notably, the MS sample exhibited superior data quality, making it the preferred choice for qualitative phase identification. Although samples displayed comparable crystalline features with large diffraction peaks’ characteristic of nanostructures, some fine distinctions are noted. MS sample diffractograms display prominent peaks at 32.6°, 35.6°, 38.7°, 48.8°, 53.7°, 58.0°, 61.6°, 66.2°, 68.0°, 72.2°, and 75.1°. These peaks, respectively, correspond to (110), (11–1), (111), (20–2), (020), (202), (−113), (022), (220), (311), and (22–2) planes, confirming the formation of the CuO monoclinic phase (JCPDS 48-1548).^30^ We used the Scherrer equation to determine crystallite sizes by averaging fwhm values based on the strongest (11–1) and (111) peaks at approximately 35.6° and 38.7°. The calculated crystallite sizes for samples ST, MS, and US are 8.7, 9.5, and 11.3 nm, respectively. Compared to the MS sample, the US sample exhibits minor peaks at 34.3°, 39.7°, and 53.2° that can be ascribed to (020), (301), and (321) peaks of the orthorhombic Cu(OH)_2_, respectively (JCPDS 13-0420).^30^ These first two peaks clearly overlap with peaks used for calculating crystallite sizes and explain the larger values found for the US sample. Owing to the high noisy-to-signal ratio of the ST sample, low-intensity diffraction peaks other than those reported for the MS sample were difficult to analyze. Therefore, the XRD analysis shows that CuO is the main crystalline phase in all samples. The US sample presented a minor secondary phase of Cu(OH)_2_, which may also form in the ST sample, but peaks are of intensity close to the background, making it difficult to draw conclusions.

XPS analyses were performed to investigate the sample surface composition. Figure S2 presents the survey, and Figure S3 shows the high-resolution C 1s spectra comparing ST, MS, and US samples. Survey spectra show the sample surfaces are relatively clean and contain peaks expected for copper oxi/hydroxides. Quantification analysis (inset, Figure S2 andTable 1) shows that the copper surface content is close to 30%, except for sample MS, which presents only ∼22%, and higher amounts of adsorbed carbon species (∼45%). It is important to note that the carbon surface content follows the trend US (28.2%) < ST (30.0%) < MS (44.8%). This surface carbon contamination is an important issue when studying electrocatalyst materials since higher adsorbed contents may deteriorate the surface-active catalytic sites. Adventitious carbon from C 1s high-resolution spectra (Figure S3) were adjusted considering four contributions with binding energy centered at 284.8, 285.9, 287.3, and 289.6 eV, corresponding to C–C (or C–H), C–OH, C=O, and O–C=O, respectively.^31,32^ These peaks were almost identical for the three samples but with different percentages, as highlighted in Table 1. The C–C peak from adventitious carbon was fixed at 284.8 eV to compensate for charging effects.

**Table 1:** XPS Component Parameters as Obtained by Best Fittings of the High-Resolution C 1s, Cu 2p, and O 1s Spectra. Total (*) Represents the Element Surface Content Obtained by Using the Survey Spectra

sample	region	component	binding energy (eV)	fwhm (eV)	area (%)	total* (%)
MS	C 1s	C–C, C–H	284.8	2.1	19.0	44.8
		C–OH	286.2		52.2	
		C=O	287.2		21.6	
		O–C=O	289.7		7.2	
	O 1s	O–Cu	528.7	2.1	60.2	32.9
		(OH)_2_^–^, (CHOO)_2_^–^	530.3		30.4	
		O–C	532.0		9.4	
	Cu 2p_3/2_	Cu_2_O	932.0	2.6	55.9	22.3
		CuO	953.4	3.0	44.1	
ST	C 1s	C–C, C–H	284.8	2.2	8.0	30.0
		C–OH	285.8		48.9	
		C=O	287.3		33.3	
		O–C=O	289.7		9.8	
	O 1s	O–Cu	528.8	2.3	49.3	40.3
		(OH)_2_^–^, (CHOO)_2_^–^	530.9		37.0	
		O–C	532.9		13.7	
	Cu 2p_3/2_	Cu_2_O	932.3	2.9	55.5	29.7
		Cu(OH)_2_	954.4	3.4	44.5	
US	C 1s	C–C, C–H	284.8	2.1	28.0	28.2
		C–OH	285.9		35.6	
		C=O	287.2		26.9	
		O–C=O	289.4		9.5	
	O 1s	O–Cu	529.1	2.5	42.1	40.4
		(OH)_2_^–^, (CHOO)_2_^–^	531.2		50.9	
		O–C	533.2		7.0	
	Cu 2p_3/2_	Cu_2_O	932.7	3.1	49.0	31.4
		Cu(OH)_2_	954.3	3.1	51.0	

**Figure 6:**
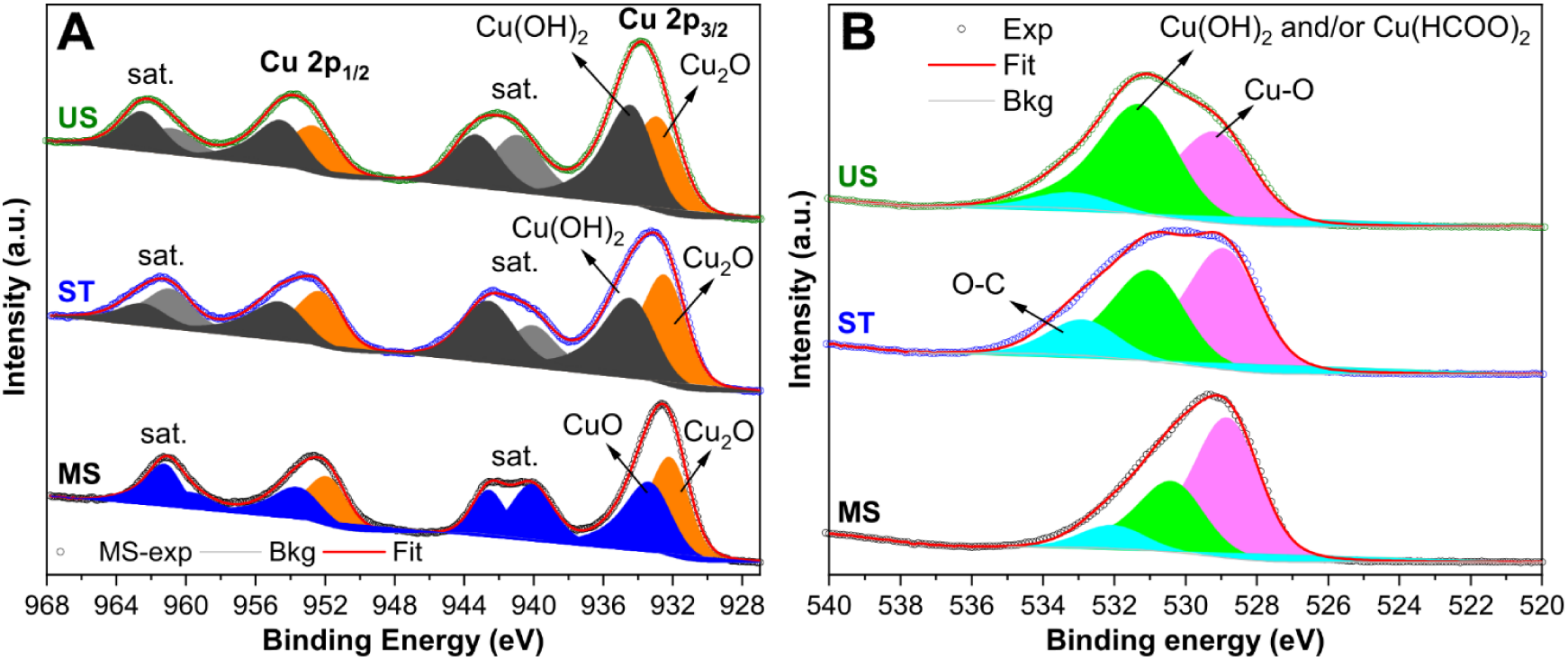
XPS high-resolution spectra of samples at the (A) Cu 2p and (B) O 1s regions.

Figure 6 shows the Cu 2p XPS spectra of these samples. We considered at least two Cu oxidation states for a reasonable fitting. MS and ST samples showed satellite peaks characteristic of CuO and Cu(OH)_2_, respectively. Therefore, we modeled them for accounting for a mixture of Cu^1+^ and Cu^2+^ contributions. Table 1 shows the quantification results. As can be seen, Cu 2p3/2 peaks at 932.0 and 933.2 eV confirmed the respective formation of Cu_2_O (55.9%) and CuO (44.1%) in the MS sample.^33,34^ Conversely, peaks at 932.4 and 934.3 eV confirmed Cu_2_O (55.5%) and Cu(OH)_2_ (44.5%) formation in the ST sample.^34,35^ Interestingly, the US presented similar contributions as for ST, with Cu 2p3/2 peaks at 932.8 and 934.3 eV, confirming the formation of Cu_2_O (49.0%) and Cu(OH)_2_ (51.0%).^35^ Cu 2p spectra fittings resulted in larger fwhm values for Cu(OH)_2_, corroborating the findings in light of the results expected from the literature.^35^

O 1s region analysis steps up the assumption of the copper species contributions. Best fittings for O 1s were obtained considering three peaks with constrained fwhm values (Figure 6). It is noteworthy that the samples present completely different O 1s features that helped to unravel the surface contribution. All spectra presented a peak in the high binding energy region, 532–533 eV, with slightly different centers. It can be assigned to O–C or surface water.^36^ Although the presence of water cannot be ruled out, this peak is in line with hydrocarbon contamination observed in the C 1s region. In the O 1s peak analysis, this O–C peak contributes 7% for the US sample, contrasting with 9% and approximately 13% for the MS and ST samples, respectively. Additionally, the O 1s spectra exhibit an O–Cu peak at 528.7, 528.8, and 529.0 eV for MS, ST, and US samples, underscoring the presence of Cu^2+^ and/or Cu^1+^ oxide formations.^34,35^ The quantification of the Cu–O peak exhibits notable variations among the samples, accounting for 60.2%, 49.3%, and 42.1% of the total surface oxygen content for MS, ST, and US samples, respectively. This trend aligns with observations in the Cu 2p region, where elevated hydroxide levels are anticipated in sample US. As hydroxide forms, the contribution of the O–Cu peak diminishes accordingly (Table 1).

Notably, to achieve a comprehensive fit for the O 1s spectra, a third peak with a binding energy in the range of 530.3–531.2 eV was employed. This further refines the analysis, providing a more detailed understanding of the oxygen species present in the samples. Peaks in this region are expected for Cu(OH)_2_ and Cu(HCOO)_2_,^31^ since the Cu 2p and C 1s previous analysis shows that both contributions may be present in the sample’s surface composition. For sample MS, as Cu 2p analysis does not suggest copper hydroxide, the O 1s peak at 530.3 eV (30.4%) is assumed to come from Cu(HCOO)_2_. Conversely, this peak positively shifts to 530.9 eV (37.0%) for the ST sample, which is relatively increasing in intensity. Since the total carbon content of these samples is less than that of MS, we assume now this peak comes from Cu(OH)_2_ formation convoluted to some Cu(HCOO)_2_ content. It is reasonable since this sample presents copper hydroxide instead of copper oxide in the Cu 2p region. For sample US, this peak is found at 531.2 eV (50.9%), which is now the majoritarian oxygen contribution due to more Cu(OH)_2_ formation, as also found in the Cu 2p region.

In summary, XPS analysis shows that the surface of the sample prepared under magnetic stirring contains a mixture of CuO and Cu_2_O and high levels of adsorbed carbon (44.8%), including the Cu(HCOO)_2_. The sample prepared under stationary conditions resulted in Cu(OH)_2_ and Cu_2_O formation, as observed for the sample under ultrasonic treatment. On the other hand, sample US showed less carbon surface contamination and more Cu(OH)_2_ species, as shown in the O 1s and Cu 2p spectra.

Building on these findings, we investigated the porosity of the materials. Figure S4 shows the Ar adsorption–desorption isotherms and the pore size distributions for the three materials. The isotherm profiles are type III, suggesting the adsorption occurs in multilayers without guaranteeing a complete first Ar monolayer, typical for mesoporous materials (Figure S4). The pore size distribution of Cu prepared by ultrasonic bath and magnetic stirring shows more volume per gram of material between 1 and 10 nm pores compared to the sample prepared stationarily. Table 2 summarizes the pore parameters. Stationary synthesis induced the highest total specific surface area (*S*_BET_) of 74 m^2^ g^–1^, while the other materials showed a total specific surface area of 70 m^2^ g^–1^. However, the external specific surface area (*S*_ext_) of such a material is the lowest, 47 m^2^ g^–1^. In comparison, Cu produced in sonication and stirring displayed 61 and 60 m^2^ g^–1^, respectively, which impacts further surface electrochemistry. The volumes of the micro (*V*_micro_) and meso (*V*_meso_) pores are similar among the samples, leading to a similar volume of pores (*V*_P_).

**Table 2:** Porous Properties Obtained from Ar Adsorption–Desorption Isotherms for Cu NPs Synthesized Under Stationary (ST), Ultrasonic Bath (US), and Magnetic Stirring (MS) Conditions^a^

	*S*_BET_ /m^2^ g^–1^	*S*_ext_ /m^2^ g^–1^	*V*_micro_ /cm^3^ g^–1^	*V*_meso_^b^/cm^3^ g^–1^	*V*_P_^*a*^ /cm^3^ g^–1^
ST	74	47	0.03	0.08	0.11
US	70	61	0.03	0.10	0.13
MS	70	60	0.03	0.11	0.14

a Volume of pores determined at saturation point.

b Volume of mesopores with pore size between 2 and 30 nm.

In summary, the materials have varied porosities but similar morphologies. Since sample ST has the lowest external specific surface area, we anticipated limited activity. The primary distinctions were discovered in surface chemical composition and crystallography. According to XRD studies, Cu NPs produced by the US have the largest crystallite (11.3 nm) and the most Cu(OH)_2_ of any sample. The results of XPS confirm that sample US contains more Cu_2_O, more Cu(OH)_2_, and less carbon contamination on its surface than the other samples.

### 3.2 Half-Cell Measurements

**Figure 7:**
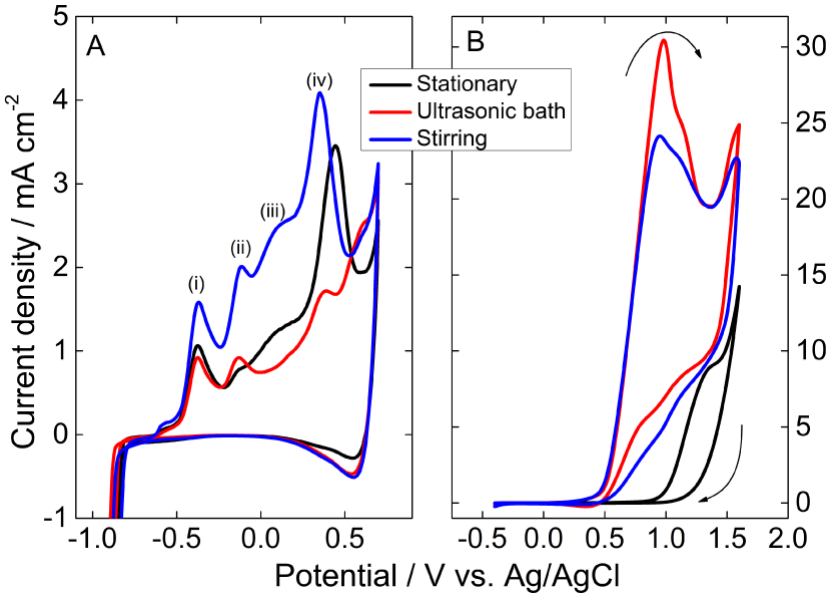
Voltammograms of the Cu NPs in 0.1 mol L^–1^ KOH at 0.05 V s^–1^ in the (A) absence and (B) presence of 0.5 mol L^–1^ methanol.

The Cu NPs were immobilized on a glassy carbon electrode and investigated in half-cells to study the electrocatalytic parameters. Figure 7A shows the electrochemical profile in an alkaline medium. Peaks (i–iv) are characteristics of the modification of the Cu oxidation state, which is better defined for the NPs synthesized by stirring. Peak (i) is a consequence of the CuOH formation, as depicted in eqs 1 and 2.^37^ Peak (ii) is ascribed to the Cu/Cu^II^ and Cu^I^/Cu^II^ transitions,^17^ detailed in eqs 3−5.^17^ Peak (iii) is due to the formation of soluble species (eq 6), parallel to the chemical reaction described in eq 7.^37^ The formation of Cu^III^ (Cu_2_O_3_) owing the products of the previous peak led to the peak (iv), as described in eqs 8 and 9.^38^ In the negative potential sweep, a cathodic peak appears at ∼0.5 V due to the partial reduction of Cu_2_O_3_. Most of the oxides are irreversible; therefore, the cathodic peaks at low potentials are not seen.
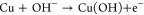
1
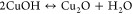
2
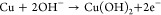
3
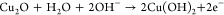
4
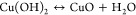
5
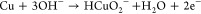
6
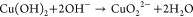
7
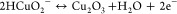
8
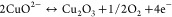
9

The complex surface phenomena in alkaline media of Cu make it difficult to directly correlate the electrooxidation of the organic to the state of the active sites since they occur parallelly. The main point is that the oxides are not deleterious for the methanol electrooxidation reaction, but Cu_2_O_3_, as seen by the ∼10-fold increase in current density at around 0.9 V onward in Figure 7B. In higher potential, when the surface becomes covered by Cu_2_O_3_, the current density from methanol electrooxidation decreases.

The onset potential of methanol electrooxidation on Cu synthesized under stationary conditions is 0.85 V, which is displaced to 0.4 V for the materials produced under convective conditions–obtained by the derivative of the voltammogram.^39,40^ The current density is also 2–3-times increased for those NPs, and those synthesized in the ultrasonic bath revealed the highest values, reaching 30.4 mA cm^–2^ (Figure 7B). The anodic peak at 0.97 V with a shoulder at 1.1 V is absent for only the Cu NPs produced stationarily.

Cu NPs produced stationarily do not show evident anodic currents during the reverse scan, while the others display two small peaks at ∼1.15 and 0.8 V. The surface reactivation is caused by the reduction of the oxides, rendering well-defined anodic currents on both forward and backward scans, as found on Pt-based catalysts for different alcohols.^41^

**Figure 8:**
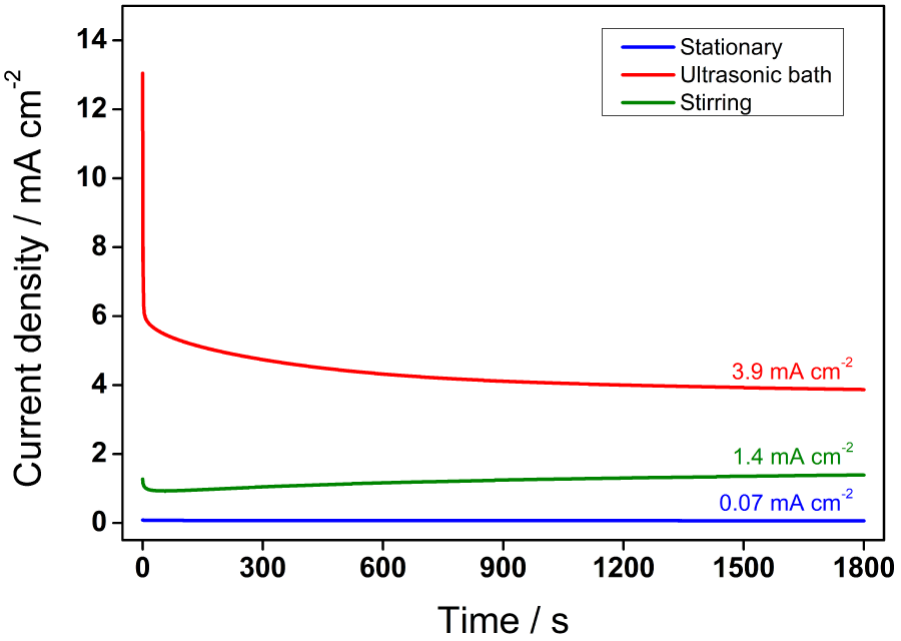
Chronoamperograms of the Cu NPs in 0.1 mol L^–1^ of KOH at 0.05 V s^–1^ for 1800 s at 0.6 V vs. Ag/AgCl.

The Cu NPs were also subjected to stationary chronoamperometry at 0.6 V, as shown in Figure 8. The potentiostatic results corroborate the potentiodynamic, suggesting that Cu NPs produced in US are the most active while those synthesized in ST are the least. Figure 8 shows that the pseudo stationary current density for the ultrasonic-assisted produced NPs is ∼55 times higher than that synthesized conventionally. At this point, it is evident that the materials display different electrocatalyses for methanol.

Since morphology is similar, crystallography and surface are key to rationalizing these results. In comparison to the other samples, US initially had a higher surface concentration of Cu(OH)_2_, which could influence the lateral Langmuir–Hinshelwood reaction between Cu(OH) and adsorbates (Cu-adsorbates, such as Cu-CO). This feature is expected at a low potential, such as 0.6 V experienced in the chronoamperometries (Figure 8). Furthermore, the sample US’s active site is more accessible due to its initial cleanliness^42^ and external surface area, demonstrated by the low carbon contaminants content on the surface, facilitating an effective surface reaction. The availability promotes dissociative adsorption, translated in high current densities in potentials higher than 0.6 V (Figure 7).

### 3.3 FTIR *In Situ* Measurements

**Figure 9:**
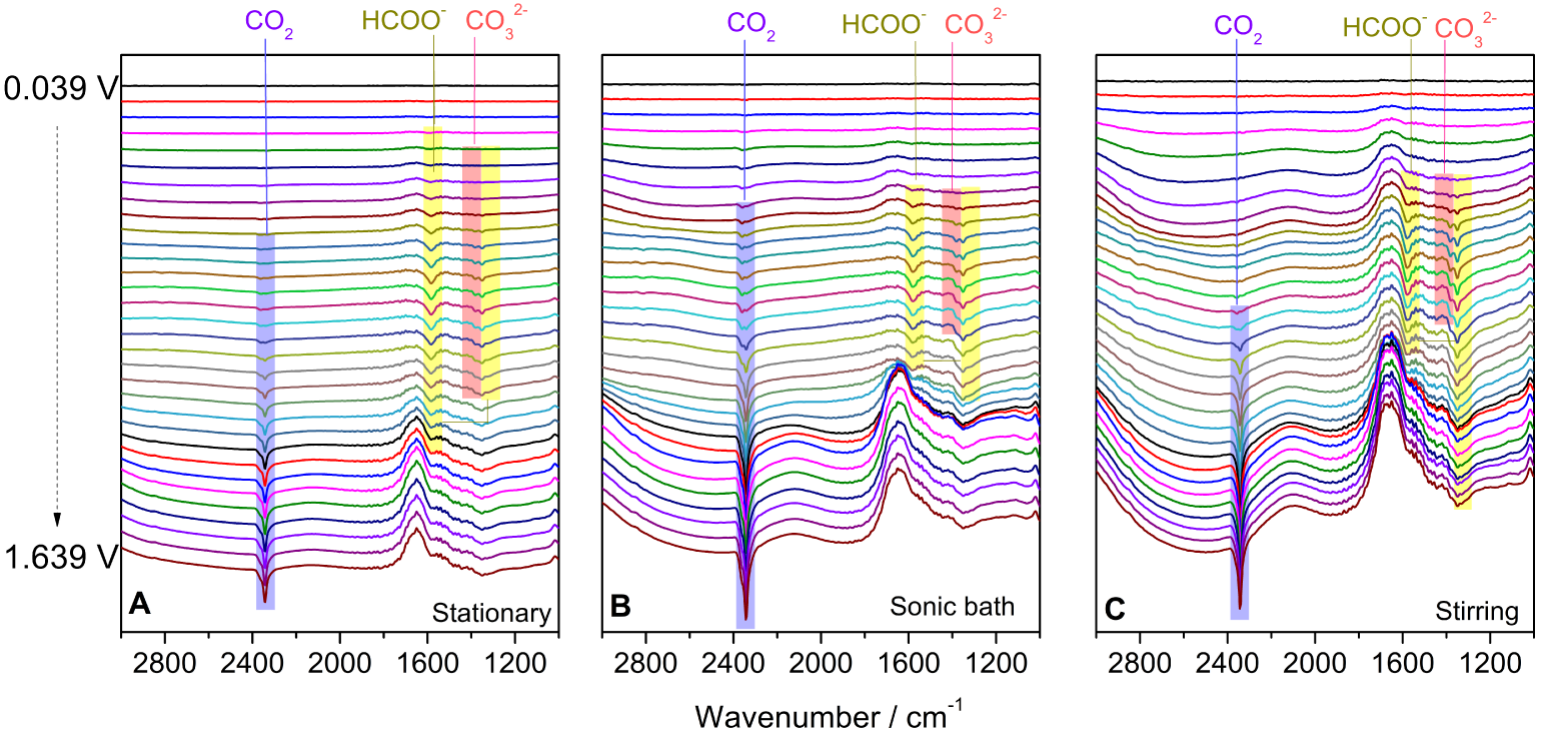
*In situ* FTIR spectra of Cu catalysts synthesized in (A) a stationary system, (B) a sonic bath, and (C) under stirring. All spectra were collected from 0.039 to 1.639 V vs. Ag/AgCl at 1 mV s^–1^ in 0.5 mol L^–1^ methanol +0.1 mol L^–1^ O_2_-free KOH.

**Figure 10:**
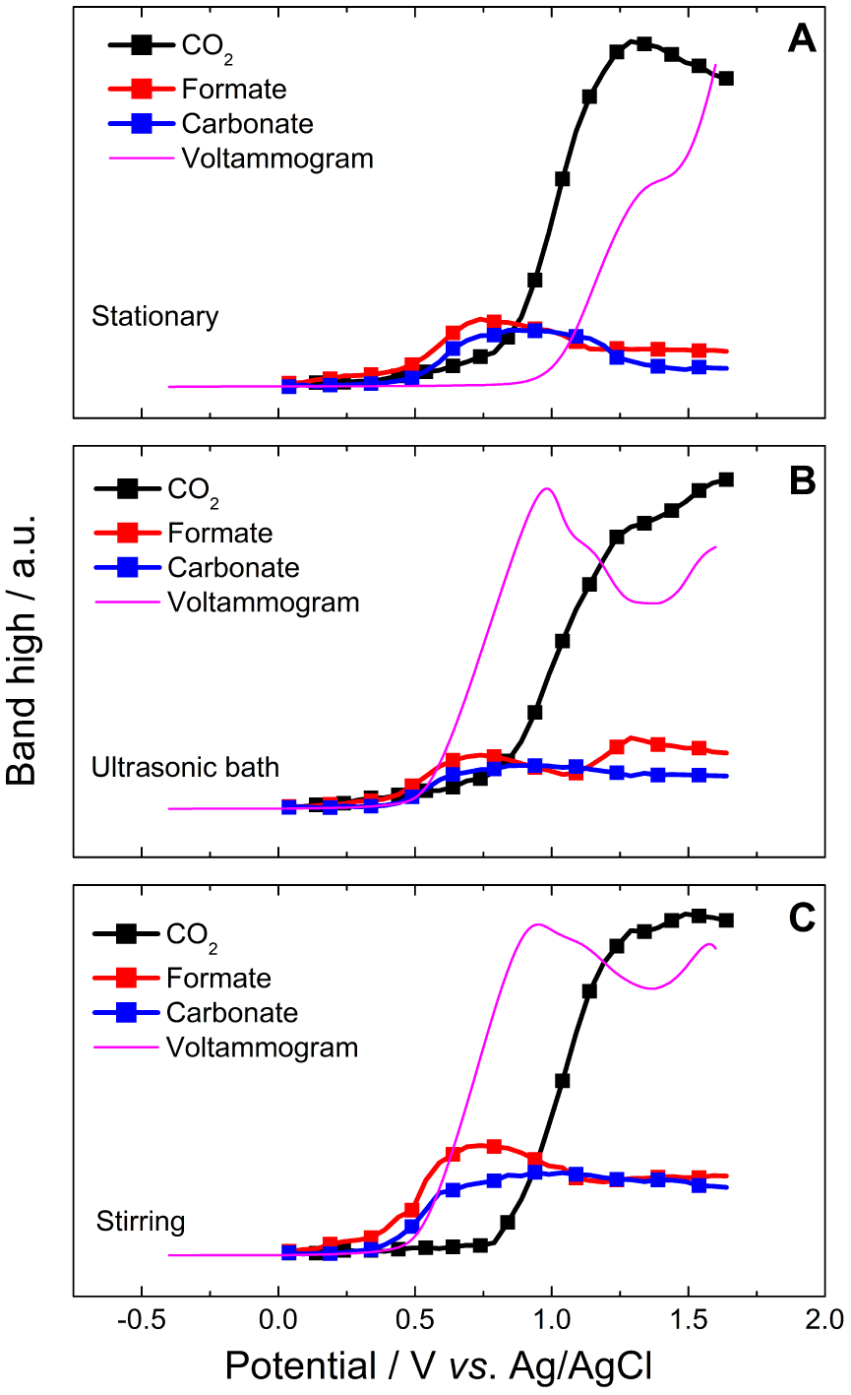
Correspondent band height results for CO_2_, formate, and carbonate from Figure 8 with the corresponding ongoing voltammograms for Cu catalysts synthesized in (A) stationary system, (B) ultrasonic bath, and (C) under stirring.

Figure 9 depicts *in situ* FTIR spectra collected during a potential scan. Main features relative to the production of CO_2_, HCOO^–^, and CO_3_^2–^ are present for all of the catalysts. Regarding species monitoring, the partial superimposition of bands hinders the proper separation of the corresponding signals. Hence, we decided to compare band heights instead of band areas. The CO_2_, formate, and carbonate results are shown in Figure 10 for all catalysts together with the corresponding ongoing voltammograms. For all cases, the CO_2_ band heights are sensibly higher than the others, but a quantitative analysis is not feasible since we do not estimate the extinction coefficients for the products. Nonetheless, since the optical conditions are nearly the same for every experiment, the relative heights for a single species can be compared among catalysts. The most interesting information is the absence of CO bands (Figure 9), suggesting that methanol electrooxidizes on Cu (and Cu oxides) to CO_3_^2–^ without producing such poisoning molecule that strongly bonds to the catalyst surface, hindering the active surface sites for further reactions.^43^ It is important to note that we are working in an alkaline medium. Hence, the CO_2_ band only appears after the pH inside the thin layer—established between the electrode and the optical window—is lowered by OH consumption.^44,45^ In other words, the OH^–^ ions present in the liquid layer between the optic window and the electrode are depleted, as they participate in the electrooxidation process. Consequently, the local pH of the solution becomes acidic, shifting the equilibrium toward the formation of CO_2_ instead of CO_3_^2–^. Therefore, the presence of CO_2_ indicates the turning point, where the pH becomes neutral or even acidic. In this condition, CO_2_ is accumulated by (a) the oxidation of methanol or other partially oxidized species or by (b) a displacement of the CO_3_^2–^— CO_2_ equilibrium triggered by the acidification of the electrolyte. In general, Figure 9 shows that methanol is electrooxidized to formate from deprotonated formic acid, to CO_3_^2–^, and inevitable to CO_2_ in a thin layer.^44^

Based on these assumptions, Figure 10A shows a unique relation between the voltammetric profile and the CO_2_ signal. Namely, the thin layer seems to be acidified before the maximum electron transfer, with the opposite behavior being observed in Figure 10B,C, i.e., in Figure 10A the thin layer pH becomes acidic (which can be inferred by the presence of CO_2_) when the currents are still very low. This apparent contradiction can be understood if we remember that formaldehyde can also be formed by the electrooxidation of methanol, particularly at low potentials, when the adsorption of OH^–^ is not favored. If that is the case, then we can assume the production of formaldehyde according to the pathway illustrated in eq 10.
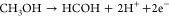
10

Note that unlike other oxidation pathways formaldehyde production involves only the release of two protons and two electrons. Those protons can, in turn, combine with OH^–^ to generate water (eq 11).
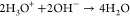
11

This last step does not involve any electrooxidation reaction. Thus, assuming that the formation of formaldehyde is favored in Figure 10A, it is possible to explain how the electrolyte pH decreases before the electron transfer rates have reached their maximum. Moreover, formaldehyde only releases 2 electrons per molecule, which justifies the low currents observed up to 1 V. Accordingly, when oxidation pathways are accelerated at high potentials, there are few OH^–^ available, and CO_2_ starts to be produced earlier.

Unfortunately, formaldehyde signals cannot be discriminated by *in situ* FTIR in the present conditions because in the presence of methanol, formaldehyde forms methoxy ethanol (CH_3_O–CH_2_–OH) and related oligomers,^46^ whose bands cannot be discriminated from the main features of the present spectra, since most of them appear in the same region of the signals we are following. Even signals appearing in different regions would probably be unnoticed, since they refer to very low concentrations.

On the other hand, if formaldehyde is not produced at high rates in Figure 9B,C, then the consumption of OH^–^ will only take place at the expense of producing formate and carbonate, which deliver 4 and 6 electrons, respectively. Accordingly, the exhaustion of OH^–^ in the thin layer will happen only when these oxidation pathways occur at high rates, which is suggested by the late CO_2_ production, in contrast with Figure 10A. This observation is in line with voltammetric and chronoamperometric results (see Figures 7 and 8, respectively).

All catalysts produce formate and carbonate at ∼0.4 V, reaching a peak at ∼0.7 V, and their consumption matches the increase in the level of CO_2_ production. This suggests that (i) formate is a parallel product of methanol electrooxidation or (ii) an intermediate since formate can be converted into CO_3_^2–^. As mentioned previously, CO_3_^2–^ formation takes place before CO_2_ since it requires pH change at the electrode–solution interface, which occurs in all three cases. However, the profiles of production and consumption of compounds are slightly different for Cu synthesized under sonication (Figure 9B).

Methanol electro-oxidation faces a second turn of events on Cu produced in a sonic bath (Figure 10B). A new formate production method starts at 1.0 V, which boosts CO_2_ production. At such high potential, the interfacial (or local) pH is already acidic; thus, formate is converted into CO_2_ directly. Hence, the band of formate increases up to 1.2 V and decreases afterward, concomitantly with a new CO_2_ increment, as shown in Figure 10B. The transfer of this additional electron is the probable reason for the improved current densities found in half-cell measurements, making Cu synthesized in a sonic bath a potential anode for methanol μFC. This stepped reaction could result from the surface composition and/or crystallography, allied with the high surface area, namely, cleanness, presence of Cu(OH)_2_, and fewer carbon contaminants.

One final aspect regarding FTIR analysis is the absence of CO as an intermediate. The Cu catalyst electrooxidizes methanol at high potential, where CO is absent. Here, we face two adverse characteristics. The absence of CO is desired since CO hinders active sites, but a high *E*_onset_ for the anodic reaction is unfeasible if it is higher than the *E*_onset_ of the cathodic reaction because Δ*G* > 0. However, using a membraneless μFC allows using a mixed-media configuration, making the use of Cu catalyst at the anode possible. To make it possible, we coupled the methanol electrooxidation on a Cu catalyst in an alkaline medium to a persulfate reduction on a metal-free carbon paper in an acidic medium.

### 3.4 Microfluidic Fuel Cell Tests

Before assembly of the μFC with the proper configuration, it is important to ensure stable colaminar. The ionic transfer at the liquids interface is stable in low R*e* (<10). The architecture used here leads to R*e* ∼ 0.6 and 1.2 for 50 and 100 μL min^–1^, respectively, as detailed in Figure S5. Moreover, we ran the μFC with blue and red diluted inks simulating the catholyte and anolyte (Figure S6) at 50 μL min^–1^. The pictures show liquids flowing through the porous electrodes and encountering the microchannel without mixing. These results guarantee efficient ionic transfer and a stable colaminar configuration. After understanding the engineering, we moved to the cell tests.

The methanol electro-oxidation reaction is facilitated on Cu NPs prepared in a sonic bath. Therefore, we assembled the μFC (Figure 1) with such anode dispersed on CP (Cu/CP) with a bare CP cathode, fed by methanol in alkaline and sodium persulfate in acidic media, respectively. Figure S7 illustrates the μFC operated here.

**Figure 11:**
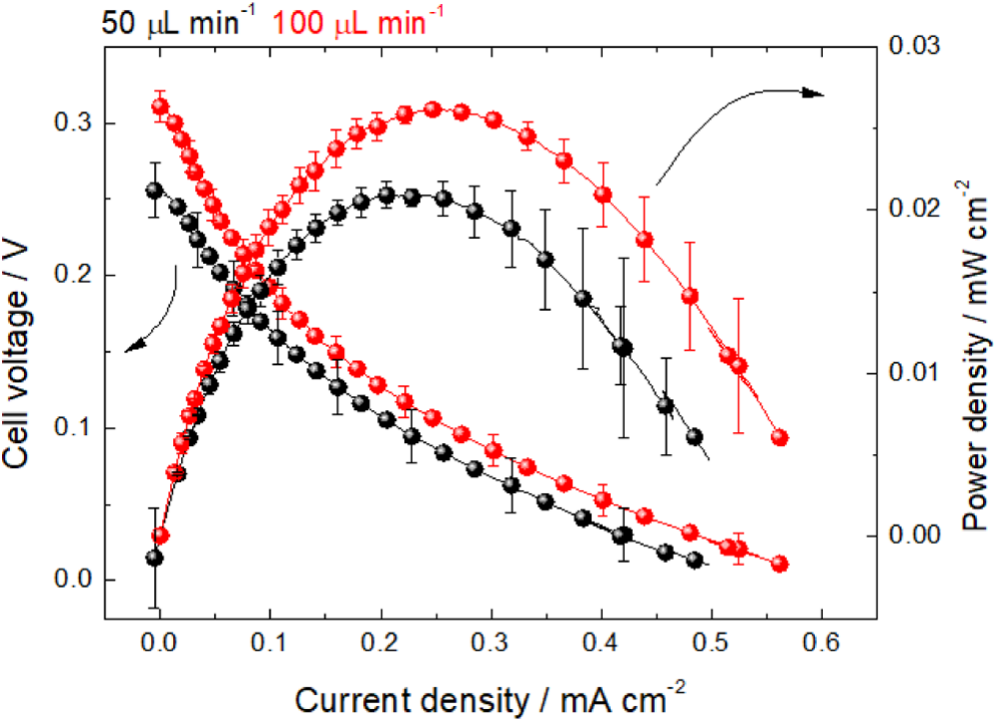
Performance of the mixed-media microfluidic, featuring polarization and power density curves for 50 (black) and 100 (red) μL min^–1^, equipped with a Cu/CP anode synthesized in an ultrasonic bath and a CP cathode. Measurements were performed with an O_2_-free 0.5 mol L^–1^ methanol +0.1 mol L^–1^ KOH as an anolyte and 1.0 mol L^–1^ Na_2_S_2_O_8_ + 1.0 mol L^–1^ H_2_SO_4_ catholyte. Polarization curves were measured from an open-circuit voltage to 0.01 V at 0.01 mV s^–1^.

Figure 11 shows the polarization and power density curves for the noble metal-free methanol μFC at 50 and 100 μL min^–1^. The onset potentials of 0.9 V for persulfate reduction on CP collected at half-cell measurements (Figure S8) allied to the 0.45 V onset for the methanol electrooxidation on Cu/CP suggest a theoretical OCV of 0.45 V and ensure spontaneous redox reaction. The polarization curves show an OCV of 0.25 and 0.3 V for 50 and 100 μL min^–1^, respectively. These values are coherent with the theoretical OCV since the flow in the microchannel leads to a stable but not perfect colaminar flow. The system is controlled by ohmic polarization, and it is not possible to identify *starving* regions at high current densities. This is because the liquid oxidant feeds the cathode at a higher rate (or equal) than the Cu/CP anode harvests electrons from methanol. The activation polarization at low current densities is intense, which is expected for an anode free of noble metals, especially containing inexpensive copper catalysts.

The maximum current densities were 0.48 and 0.56 mA cm^–2^ for 50 and 100 μL min^–1^. The maximum power density increases from 20 to 26 μW cm^–2^ at 50 and 100 μL min^–1^, respectively. The results are reproducible but show a deviation of ∼0.0087 mW cm^–2^ in current densities higher than 0.4 mA cm^–2^. The conversion of methanol into carbonate passing by formate and into formate itself through the porous electrode without CO poisoning is key to making Cu NPs synthesized in the sonic bath a good anode. It is worth noticing that the reaction on bulk solution leads to formate and carbonate, but CO_2_ may be found in the porous electrodes whether the residence time is increased, such as in 1 μL min^–1^ or passive flow. The interfacial pH is unlikely to change to acidic at 50 and 100 μL min^–1^.

At least to our knowledge, this is the first non-noble anode and metal-free cathode assembled in a methanol μFC. Generally, metal-free catalysts are enzymatic, showing selective reaction rendering up to 24 μW cm^–2^, but using complex and hard-to-produce cathode, like the mixture of laccase, anthracene-modified multiwall carbon nanotubes, and tetrabutylammonium bromide-modified Nafion (MWCNTs/laccase/TBAB-Nafion).^47^ Most of the works deal with noble metal anodes.^13,48 −51^

Besides the doubtless advantage of opening the possibility for using a Cu-based anode and metal-free cathode to convert energy from an alcohol, a decrease in cost of power is also relevant. Zanata et al. proposed a cost normalization approach to analyze whether the use of a catalyst is adequate or not, as a complementary method.^52^ The main idea is to find the output power by mass of the main catalyst at the anode to finally convert the cost of such an anode, revealing the cost of power [mW US$^–1^]. To achieve that, we performed thermogravimetry for all Cu materials and for a Pt/C/CP anode, used as a reference, as shown in Figure S9. The reference catalyst was built using 60% Pt/C on CP prepared by the same method.^26^ The loadings of Cu on CPs in comparison to a Pt/C/CP are shown in Table S1. The Cu percentages on CP are 4.65%, 2.22%, and 1.30% for the synthesis ST, MS, and US, respectively. This evidence that the ultrasonic bath induces more dispersed NPs, capable of delivering more current with lower loading.

We converted the maximum power density into the mass power [mW mg^–1^] to find the cost of power based only on the price of the anode, the object of this study (Table S2). Even though the comparison is always unfair due to the differences in terms of reactants and catalysts, Table S2 shows that although the mass power can be low, the cost of Cu is so insignificant that such an anode may be of interest to deliver power. For instance, Lan et al. built a μFC equipped with a noble-metal based Pd/C anode and a CP cathode fed by formate and Na_2_S_2_O_8_ with a cost of 825.70 mW US$^–1^.^25^ Here, using the Cu/CP anode and CP cathode, the conversion reached 274.00 mW US$^–1^. Noble metal catalysts deliver more energy even considering their prices, but this work shows that it is possible to build a galvanic cell with very low cost, and still deliver useful power. Thus, modifying the Cu anode may be an interesting strategy, equivalent to that being used more decades with Pt, but starting with a very low cost.

In summary, we showed a fast method for reduced scale synthesis of Cu materials capable of electrooxidizing methanol to carbonate. The use of the Cu anode avoids CO poisoning due to its catalytic properties and the high applied potential. Using such an anode in a conventional all-alkaline (or all-acidic) fuel cell is not feasible because the cell voltage would be close to zero or even negative when coupled to an oxidant reduction, making the overall reaction nonspontaneous. A mixed-media configuration allowed us to build an inexpensive Cu anode coupled to a metal-free cathode in a methanol μFC. We used a simple Cu anode for proof-of-concept, but the μFC may benefit further by alloying it with other inexpensive metals or by adding cocatalysts. The single cell can be scaled out to improve output power and could be disposable due to its low cost.

## 4 Conclusions

We propose a simple, fast, and small-scale room-temperature synthesis of Cu NPs. The syntheses were conducted under stationary conditions (ST), under mechanical stirring (MS), and under sonication (US). The obtained surfactant-free NPs are readily catalytically active without the need for activation. The US-synthesized sample had more Cu_2_O and Cu(OH)_2_ on the surface than the other samples. These Cu NPs also have a high specific surface area and reduced carbon impurities on their surface. The most likely cause of the increased cleanliness and partial reduction of oxides into Cu(OH)_2_ is the local heating caused by the sonic bath.

FTIR *in situ* analysis showed that methanol is electrooxidized to formate and to CO_3_^2–^. The fast OH^–^ consumed in the thin layer of the spectroelectrochemical cell turns the pH acidic, leading to the formation of CO_2_ formation instead. Also, the absence of CO bands for all catalysts was observed, suggesting that methanol electrooxidizes on Cu (and Cu oxides) to CO_3_^2–^ without passing through such a poisoning molecule that strongly bonds to the catalyst surface, hindering the active surface sites for further reactions. Another hypothesis is that the formation and consumption of CO show fast kinetics on the Cu surface at high potentials.

Among the Cu catalysts produced by the different methods, Cu NPs synthesized under an ultrasonic bath were the most active for electrooxidation of methanol in an alkaline medium. Half-cell measurements reveal an *E*_onset_ of 0.45 V for the methanol electrooxidation, and high current density, which allied to the high conversion into carbonate without the presence of CO, makes this material a potential anode. The main reason for such an improved reaction is the high external specific surface area, surface cleanliness, and presence of more Cu(OH)_2_ that may react with adsorbates, increasing current density and making more active sites available. This anodic reaction was coupled to sodium persulfate reduction in an acidic medium, displaying 0.9 V of *E*_onset_, leading to 0.45 V of theoretical OCV. Experimentally, the noble metal-free methanol μFC showed 0.25 V of OCV, 0.56 mA cm^–2^ of maximum current density, and 26 μW cm^–2^ at 100 μL min^–1^.

Therefore, we showed a small-scale method to synthesize active Cu NPs for methanol electrooxidation. We reported a rational strategy to understand the activity of the material from fundamental spectroelectrochemistry to energy conversion. This first assembled noble metal-free anode and metal-free cathode may benefit further from using high conductive electrolytes, cocatalysts, and high flow rates.

## Supporting Information Available

Pictures of the steps of the Cu nanoparticle synthesis, voltammograms of carbon paper in the presence and absence of sodium persulfate, XPS survey, Ar isotherms, calculations of Reynolds number, and thermogravimetric experiments (PDF)
