# Supplementary material for: A Comprehensive Investigation of Methanol Electrooxidation on Copper Anodes: Spectroelectrochemical Insights and Energy Conversion in Microfluidic Fuel Cells
Source: ACS Appl Mater Interfaces. 2024 Jun 25;16(27):35255–67. doi: 10.1021/acsami.4c08472 (PMC11247425; doi:10.1021/acsami.4c08472)
Supplement: Supplementary file 1 — am4c08472_si_001.pdf [file am4c08472_si_001.pdf]

Supporting Information

A comprehensive investigation of methanol electrooxidation on copper anodes: Spectroelectrochemical insights and energy conversion in microfluidic fuel cells

Breno D. Queiroz,[a] Pedro-Lucas S. Vital,[a] Kaê O. Budke,[a] Natalia Rey-Raap,[b] Ana Arenillas,[b] Guilherme M. O. Barra,[c] Dênis S. Ferreira,[d] Giuseppe A. Camara,[d] Heberton Wender,*[a] Cauê A. Martins,*[a]

[a]Institute of Physics, Universidade Federal de Mato Grosso do Sul, CP 549, 79070-900, Campo Grande, MS, Brazil.

[b]Group MATENERCAT, Instituto de Ciencia y Tecnología del Carbono, INCAR-CSIC, Francisco Pintado Fe 26, 33011 Oviedo, Spain

[c]Departamento de Engenharia Mecânica, Universidade Federal de Santa Catarina, 88040-900, Florianópolis, Brazil.

[d]Institute of Chemistry, Universidade Federal de Mato Grosso do Sul, CP 549, 79070-900, Campo Grande, MS, Brazil.

*heberton.wender@ufms.br, and *caue.martins@ufms.br

---

* Corresponding Author. Phone: +55 67 99262 4202

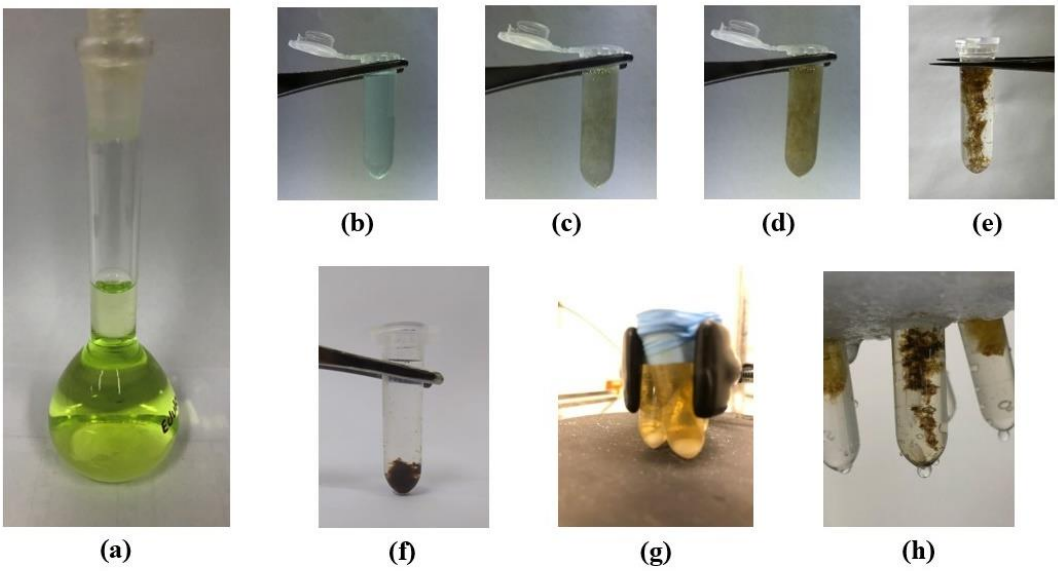

Figure S1. Pictures of the reduced scale synthesis of Cu nanoparticles over time, featuring (a) the metallic precursor H_2CuCl_4, (b) the beginning of the reaction, and after (c) 20 min, and (d) 40 min. (e) shows the formation of particles and (f) the material after synthesis in a stationary condition. (g) shows the synthesis in magnetic stirring and (h) the material after synthesis in an ultrasonic bath. The stationary, the stirred, and the assisted-in ultrasonic bath dispersions are placed in an ultrasonic bath for a few seconds for homogenization before electrode preparation, assuming a limpid dispersion such as figure (g).

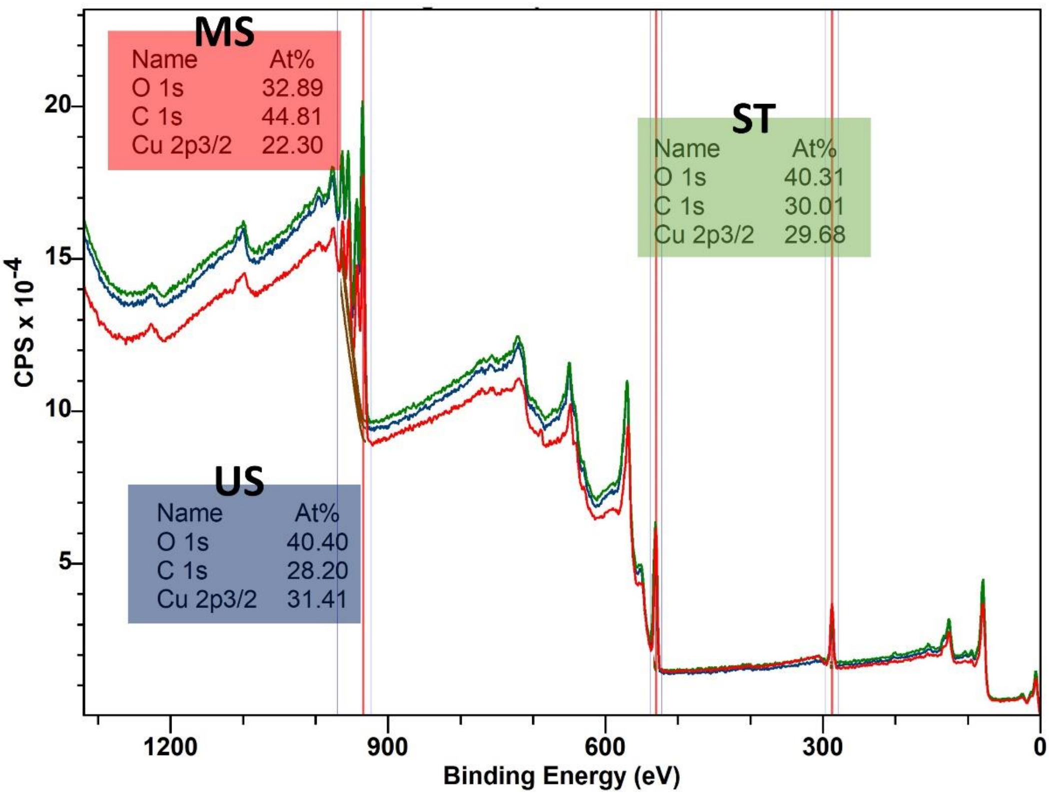

Figure S2. XPS survey spectra with quantification data.

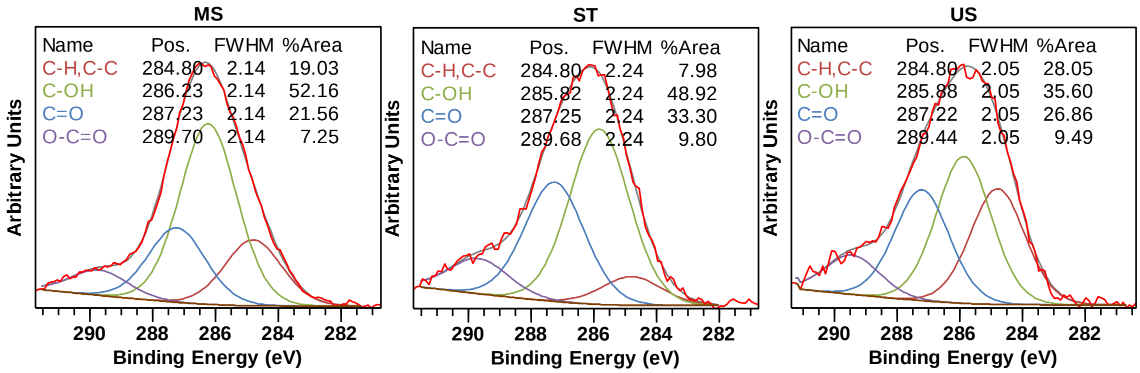

Figure S3. C 1s high-resolution spectra of MS, ST, and US samples, and the respective component fitting parameters.

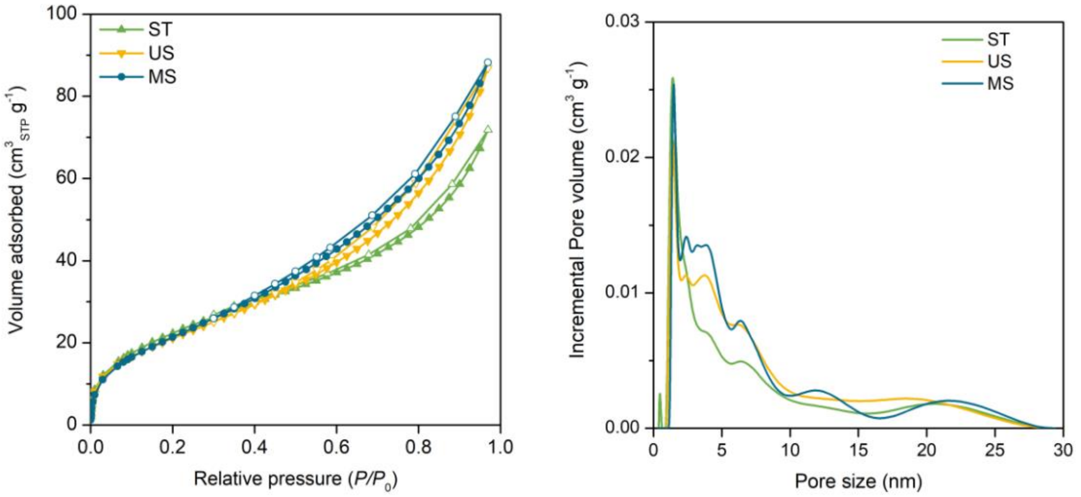

Figure S4. Ar adsorption-desorption isotherms (left) and pore size distribution (right) for Cu nanoparticles synthesized in stationary (ST), ultrasonic bath (US), and magnetic stirring conditions, as indicated in the figure.

Calculation of Reynolds' number based on fluid density ($\rho = 997\ kg\ m^{-3}$), average velocity ($U = Q/A$), hydraulic diameter (D_h) and dynamic viscosity ($\mu = 8.9\ 10^{-4}\ kg\ m^{-1}\ s^{-1}$), where A is the cross section of the microchannel, given by the product of its width (w) by its height (h).

$$Q = 50\mu L\ min^{-1} = 8.33\ 10^{-10}\ m^3\ s^{-1}$$
$$Q = 100\mu L\ min^{-1} = 1.66\ 10^{-9}\ m^3\ s^{-1}$$

$$A = w \times h = 0.003 \times 0.0002 = 6\ 10^{-7}\ m^2$$

$$D_h = \frac{2 \times w \times h}{(w+h)} = \frac{2 \times 0.003 \times 0.0002}{(0.003 + 0.0002)} = 3.75\ 10^{-4}\ m$$

$$U_{50} = \frac{Q}{A} = \frac{8.33\ 10^{-10}}{6\ 10^{-7}} \rightarrow U_{50} = 1.388\ 10^{-3}\ m\ s^{-1}$$
$$U_{100} = \frac{Q}{A} = \frac{1.66\ 10^{-9}}{6\ 10^{-7}} \rightarrow U_{100} = 2.777\ 10^{-3}\ m\ s^{-1}$$

$$Re = \frac{\rho U D_h}{\mu}$$

$$Re_{50} = \frac{997 \times 1.388\ 10^{-3} \times 3.75\ 10^{-4}}{8.9\ 10^{-4}} \rightarrow Re_{50} = 0.583$$

$$Re_{100} = \frac{997 \times 2.777\ 10^{-3} \times 3.75\ 10^{-4}}{8.9\ 10^{-4}} \rightarrow Re_{50} = 1.167$$

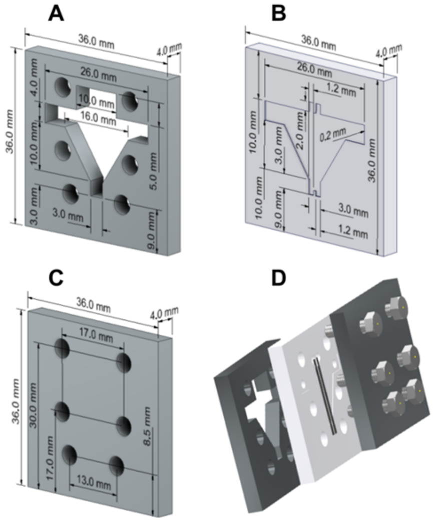

Figure S5. Calculation of Reynolds' number (R*e*) for 50 and 100 µL min^{-1} based on fluid density (ρ), average velocity (U), hydraulic diameter (D_h), and dynamic viscosity. Besides the calculation, the cell dimensions are detailed in (a-d).

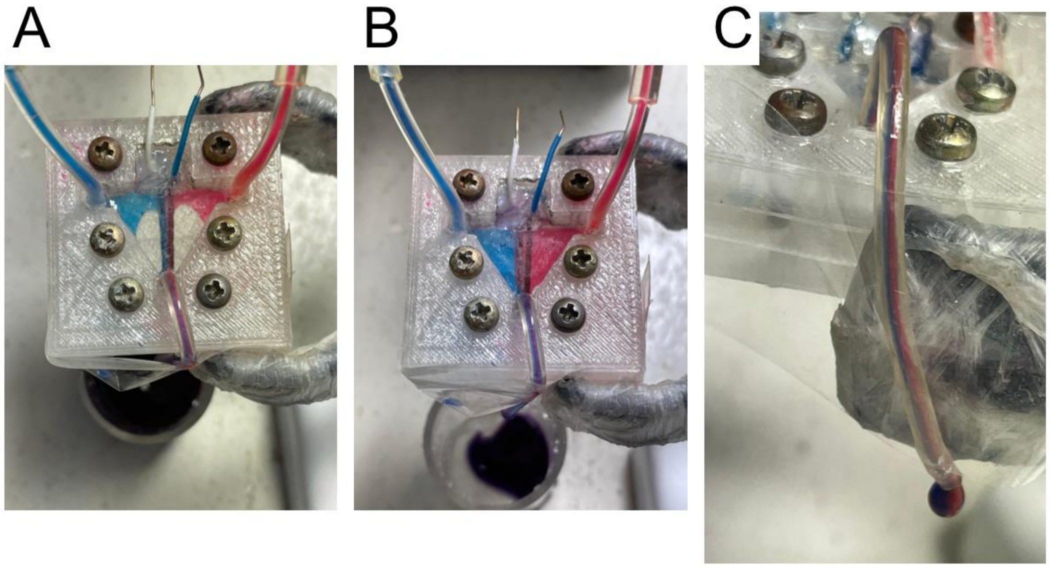

Figure S6. Photography of the microfluidic fuel cell fed by inks at 50 µL min^{-1}, featuring (a) the beginning and (b) the moment the colaminar microchannel is built. (c) illustrative photography of the two-color stable colaminar flow at the outlet.

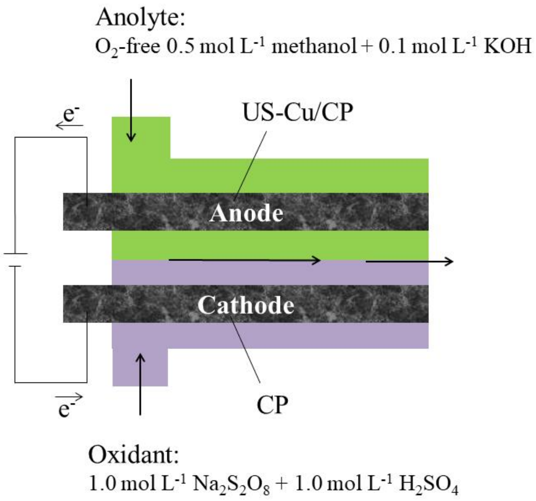

Figure S7. Illustrative scheme of the µFC functioning. The cell has a Cu/CP from NPs synthesized in an ultrasonic bath node and a bare CP as cathode, fed by the anolyte and catholyte indicated in the figure.

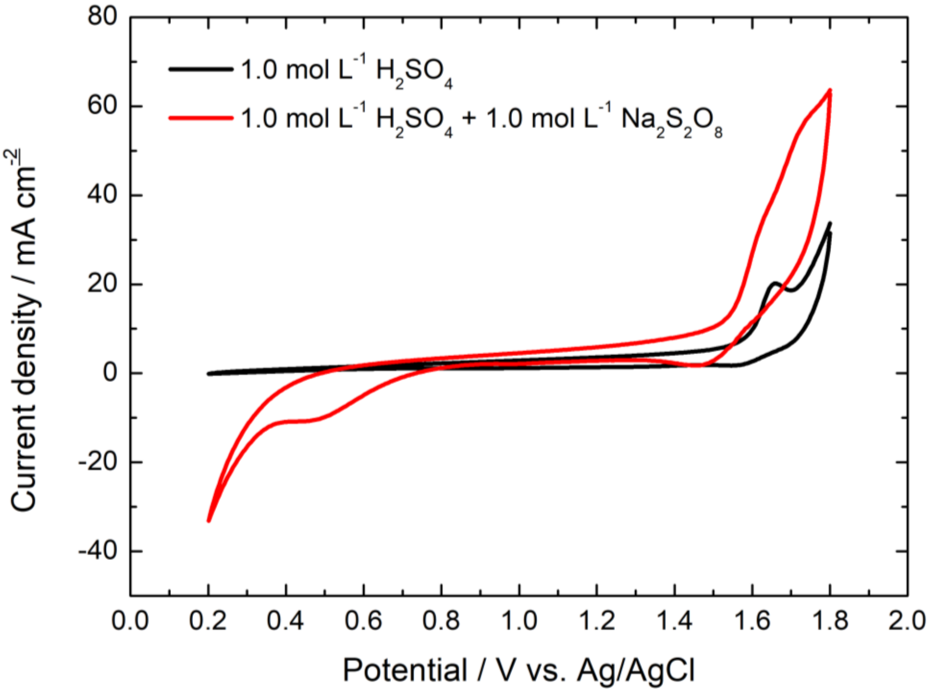

Figure S8. Cyclic voltammograms of bare carbon paper in 1.0 mol L^{-1} H_2SO_4 at 0.05 V s^{-1} in the presence and absence of 1.0 mol L^{-1} $Na_2S_2O_8$.

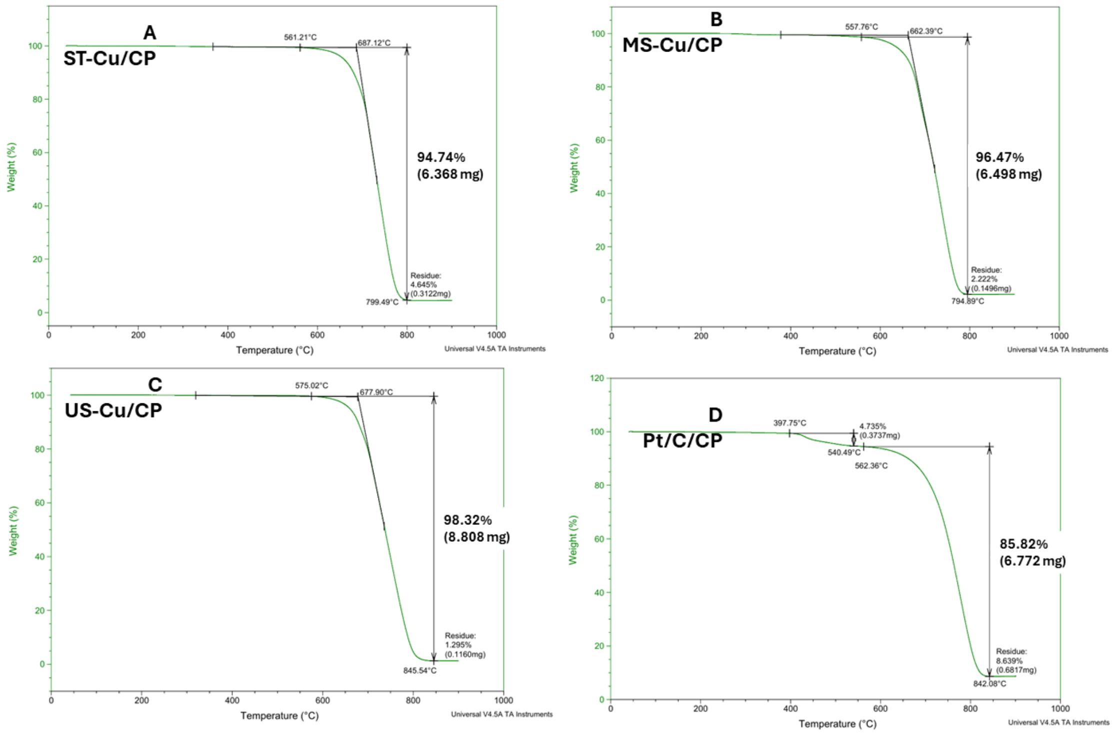

Figure S9. Thermograms of (a) ST (b) MS, and (c) US synthesized Cu nanoparticles. Featuring (d) thermogram of Pt/C/CP used by Guima et al.[1]

Table S1. Loadings of metals on carbon paper calculated by thermogravimetry.

Catalyst	Mass of metal / mg	% of metal on CP
ST Cu/CP	0.0520	4.65%
MS Cu/CP	0.0374	2.22%
US Cu/CP	0.0193	1.30%
57% Pt/C/CP	0.1704	8.64%

Table S2. Perfomance parameters of microfluidic fuel cells.

Reference	Flow rate/µL min^{-1}	Maximum power density/ mW cm^{-2}	Maximum mass density / mW mg^{-1}	*Price / US$ per mg	Cost of power/ mW $US\$^{-1}$	Anode	Anolyte	Cathode	Catholyte
[2]	100	-	13.1	**0.00236	5,5546.56	Ru@Pt NP on Graphene Oxide + CNT aerogel	1M MeOH + 1M KOH	Pt/C	1M KOH (air)
[3]	100	-	10.15	**0.00236	4,297.53	Ru@Pt NP on Graphene Oxide + CNT aerogel	1M MeOH + 1M KOH	Pt/C	1M KOH (air)
[4]	5	-	9.39	0.00236	3,975.74	Pt NP on Graphene Oxide aerogel	1M MeOH + 1M KOH	Pt/C	1M KOH (air)
[5]	200	90	30	0.3633	825.70	Pd/CP	0.5M HCOONa + 2M NaOH	CP	0.5M $Na_2S_2O_8$ + 1M H_2SO_4
[1]	100	34.29	***3.02	0.00236	1,277.84	Pt/C/CP	1M Glycerol + 1M KOH	CP	0.5M $Na_2S_2O_8$ + 1M H_2SO_4
[1]	100	28.18	***2.48	0.00236	1.050,14	Pt/C/CP	1M Glycerol + 1M KOH	CP	Bleach + 1M H_2SO_4
This work	50	0.02	0.002358	0.000011	210.77	Cu/CP	1M MeOH + 1M KOH	CP	M $Na_2S_2O_8$ + 1M H_2SO_4
This work	100	0.026	0.003066	0.000011	274.00	Cu/CP	1M MeOH + 1M KOH	CP	M $Na_2S_2O_8$ + 1M H_2SO_4

*Calculated considering the price in the stock exchange in may 2024.

**Only Pt was considered. We were not able to calculated the mass of Ru.

***The loading was calculated here.

References

(1) Guima, K.-E.; Zanata, C. R.; Martins, C. A. Exploring Liquid Oxidants and Metal-Free Cathode for Enhanced Performance in a Reusable 3D-Printed Glycerol Microfluidic Fuel Cell. *Electroanalysis* ***2024***, *36*, e202300223. https://doi.org/10.1002/elan.202300223.

(2) Kwok, Y. H.; Wang, Y. F.; Tsang, A. C. H.; Leung, D. Y. C. Graphene-Carbon Nanotube Composite Aerogel with Ru@Pt Nanoparticle as a Porous Electrode for

Direct Methanol Microfluidic Fuel Cell. *Appl. Energy* **2018**, *217*, 258–265. https://doi.org/10.1016/j.apenergy.2018.02.141.

(3) Kwok, Y. H.; Wang, Y. F.; Tsang, A. C. H.; Leung, D. Y. C. Ru@Pt Core Shell Nanoparticle on Graphene Carbon Nanotube Composite Aerogel as a Flow through Anode for Direct Methanol Microfluidic Fuel Cell. *Energy Procedia* **2017**, *142*, 1522–1527. https://doi.org/10.1016/j.egypro.2017.12.602.

(4) Kwok, Y. H.; Tsang, A. C. H.; Wang, Y.; Leung, D. Y. C. Ultra-Fine Pt Nanoparticles on Graphene Aerogel as a Porous Electrode with High Stability for Microfluidic Methanol Fuel Cell. *J. Power Sources* **2017**, *349*, 75–83. https://doi.org/10.1016/j.jpowsour.2017.03.030.

(5) Lan, Q.; Ye, D.; Zhu, X.; Chen, R.; Liao, Q.; Zhang, T.; Zhou, Y. Direct Formate/Persulfate Microfluidic Fuel Cell with a Catalyst-Free Cathode and High Power Density. *ACS Sustain. Chem. Eng.* **2021,** *9*, 5623-5630. https://doi.org/10.1021/acssuschemeng.1c00395.
